# Contribution of Host miRNA-223-3p to SARS-CoV-Induced Lung Inflammatory Pathology

**DOI:** 10.1128/mbio.03135-21

**Published:** 2022-03-01

**Authors:** Lucía Morales, Juan Carlos Oliveros, Luis Enjuanes, Isabel Sola

**Affiliations:** a Department of Molecular and Cell Biology, National Center of Biotechnology (CNB-CSIC), Madrid, Spain; b Computational Genomics Service, National Center of Biotechnology (CNB-CSIC), Madrid, Spain; Virginia Polytechnic Institute and State University

**Keywords:** coronavirus, SARS-CoV, micro RNAs, virus-host interaction, lung inflammatory pathology, RNAseq, NLRP3, *CFTR*

## Abstract

Severe acute respiratory syndrome coronavirus (SARS-CoV) and the closely related SARS-CoV-2 are emergent highly pathogenic human respiratory viruses causing acute lethal disease associated with lung damage and dysregulated inflammatory responses. SARS-CoV envelope protein (E) is a virulence factor involved in the activation of various inflammatory pathways. Here, we study the contribution of host miRNAs to the virulence mediated by E protein. Small RNAseq analysis of infected mouse lungs identified miRNA-223 as a potential regulator of pulmonary inflammation, since it was significantly increased in SARS-CoV-WT virulent infection compared to the attenuated SARS-CoV-ΔE infection. *In vivo* inhibition of miRNA-223-3p increased mRNA levels of pro-inflammatory cytokines and *NLRP3* inflammasome, suggesting that during lung infection, miRNA-223 might contribute to restrict an excessive inflammatory response. Interestingly, miRNA-223-3p inhibition also increased the levels of the *CFTR* transporter, which is involved in edema resolution and was significantly downregulated in the lungs of mice infected with the virulent SARS-CoV-WT virus. At the histopathological level, a decrease in the pulmonary edema was observed when miR-223-3p was inhibited, suggesting that miRNA-223-3p was involved in the regulation of the SARS-CoV-induced inflammatory pathology. These results indicate that miRNA-223 participates in the regulation of E protein-mediated inflammatory response during SARS-CoV infection by targeting different host mRNAs involved in the pulmonary inflammation, and identify miRNA-223 as a potential therapeutic target in SARS-CoV infection.

## INTRODUCTION

Severe acute respiratory syndrome coronavirus (SARS-CoV) emerged in China in 2002 and spread to more than 30 countries, infecting around 8,000 people and causing the death of 10–50% infected individuals, depending on the age (1–3). Bats were identified as the reservoirs of SARS-like coronaviruses (CoVs) (4), indicating that the emergence of new CoVs potentially pathogenic for humans was a likely event. Indeed, a novel human respiratory CoV, Middle East Respiratory Syndrome CoV (MERS-CoV), emerged in 2012 in Saudi Arabia (5), infecting ever since more than 2,590 people and causing the death of 940, with a mortality ∼35% (https://www.ecdc.europa.eu/en/middle-east-respiratory-syndrome-coronavirus). The emergence of SARS-CoV-2 in December 2019, leading to the current pandemic of coronavirus disease 2019 (COVID-19), with more than 180 million people and more than 4 million deaths as of early July 2021, has caused an unprecedented disruption in human society, underscoring the need for effective prevention and therapeutic strategies. Understanding the mechanisms involved in CoV virulence and virus–host interactions has become a priority for the rational design of effective antiviral therapies against known and future emergent CoVs.

A dysregulated innate immune response resulting in elevated lung cytokine and chemokine levels is a main determinant of SARS-CoV (6) and SARS-CoV-2-induced pathogenesis (7). Our group has described that SARS-CoV envelope (E) protein is a virulence factor (8–11) contributing to SARS-CoV-induced lung inflammatory pathology by different mechanisms. E protein activates the NF-κB dependent proinflammatory response (12) and induces the p38 mitogen-activated protein kinase (MAPK) pathway mediated by the interaction of E protein PDZ-binding motif with syntenin PDZ domain (13). Furthermore, E protein ion channel activity participates in the transport of Ca^2+^ in infected cells, which activates NLRP3 inflammasome and production of IL-1ß (14), a main contributor to severe inflammation that characterizes the acute respiratory distress syndrome (ARDS) observed in SARS-CoV and SARS-CoV-2 patients (15–17).

MicroRNAs (miRNAs) are small noncoding RNAs (ncRNAs) around 22 nucleotides (nt) in length that direct posttranscriptional regulation of mRNA expression, mostly by repressing, but sometimes by stimulating, mRNA translation (18, 19). In humans and other mammals, miRNAs fine-tune the expression of most mRNAs, essentially involved in all developmental, physiological and pathological processes, including the response to infections. miRNAs are processed from a long primary transcript by the sequential cleavage of two endonucleases, Drosha, in the nucleus, and Dicer, in the cytoplasm, leading to 22 nt RNA duplexes. miRNA duplexes are loaded into an Argonaute protein included in RNA induced silencing complexes (RISC) (20), in which the mature miRNA pairs to complementary sites within mRNAs to direct their posttranscriptional repression, predominantly by decreasing target mRNA levels (21, 22). The most common sites for base-pairing the miRNA “seed sequence” (nt 2–7) are located at the 3′ UTR of target mRNAs (23). miRNAs have a moderate impact on mRNA expression levels (1.2- to 4-fold), leading to fine-tuning gene expression (24). However, the presence of multiple miRNA sites on the same target mRNA may act cooperatively, leading to a more substantial repression (25). Since one miRNA can be targeting several mRNAs and the same mRNA can be regulated by different miRNAs (26), miRNAs provide a modular and combinatorial system integrated within complex regulatory networks (27). Posttranscriptional regulation by miRNAs confers an additional layer of complexity in the regulation of gene expression, which is essential to cope with environmental stresses, infections, and diseases. During the inflammatory response, miRNAs are coregulated with protein-coding genes. The specific time frame of miRNAs biogenesis and their mechanism of action may confer unique regulatory properties to modulate the magnitude of the response (24, 28). Certain miRNAs participate in negative feedback loops, whereas others contribute to amplify the response by repressing inhibitors (29).

Little is known about the contribution of miRNAs to the regulation of acute inflammation induced by viral infections. Aguado and colleagues described that host miRNAs regulate the expression of pro-inflammatory cytokines late in the innate immune response against viral infections (30). Differential expression of miRNAs in the lungs of several mouse strains infected with SARS-CoV suggested that miRNAs might play a regulatory role in the host response to SARS-CoV (31). Since the biological effects of miRNAs are highly cell-type and temporal-stage specific (21), it is critical to study miRNA functions *in vivo*, in a biologically relevant system, in which miRNAs and their target mRNAs are expressed at physiological levels (32).

This work addresses the relevance of miRNAs in SARS-CoV pathogenesis by studying the differential expression of host miRNAs in the lungs of mice infected with the virulent SARS-CoV-WT or the attenuated mutant without the E gene (SARS-CoV-ΔE). Small RNA-sequencing (RNAseq) revealed 23 differentially expressed annotated miRNAs in virulent versus attenuated SARS-CoV infection, representing potential regulators of pulmonary inflammation induced by E protein. Functional enrichment analysis of miRNA-regulated pathways identified miRNA-223-3p, which was overexpressed in the virulent infection, as a regulator of cytokine-mediated inflammation. Inhibition of miRNA-223-3p *in vivo* with antisense locked nucleic acids (LNAs) reduced lung alveolar edema, which is a major sign of SARS-CoV-induced pulmonary inflammation. As a result of miRNA-223 inhibition, mRNA expression levels of NLRP3 inflammasome and pro-inflammatory chemokines CXCL10, CXCL2, and IL-1ß were increased, suggesting a contribution of miR-223-3p *in vivo* to limit excessive lung inflammation induced by SARS-CoV infection. miRNA-223 inhibition also increased the expression of ion transporter cystic fibrosis transmembrane regulator (*CFTR*), involved in the resolution of alveolar edema (33–35), which was significantly reduced in the lungs of mice infected with the virulent virus. Thus, we describe that miRNA-223 participates *in vivo* in the regulation of SARS-CoV-induced lung inflammation by affecting not only the expression of pro-inflammatory cytokines and chemokines, but also cell factors associated with edema resolution.

## RESULTS

### miRNAs differentially expressed in the lungs of mice infected with attenuated SARS-CoV-ΔE or virulent SARS-CoV-WT.

**FIG 1:**
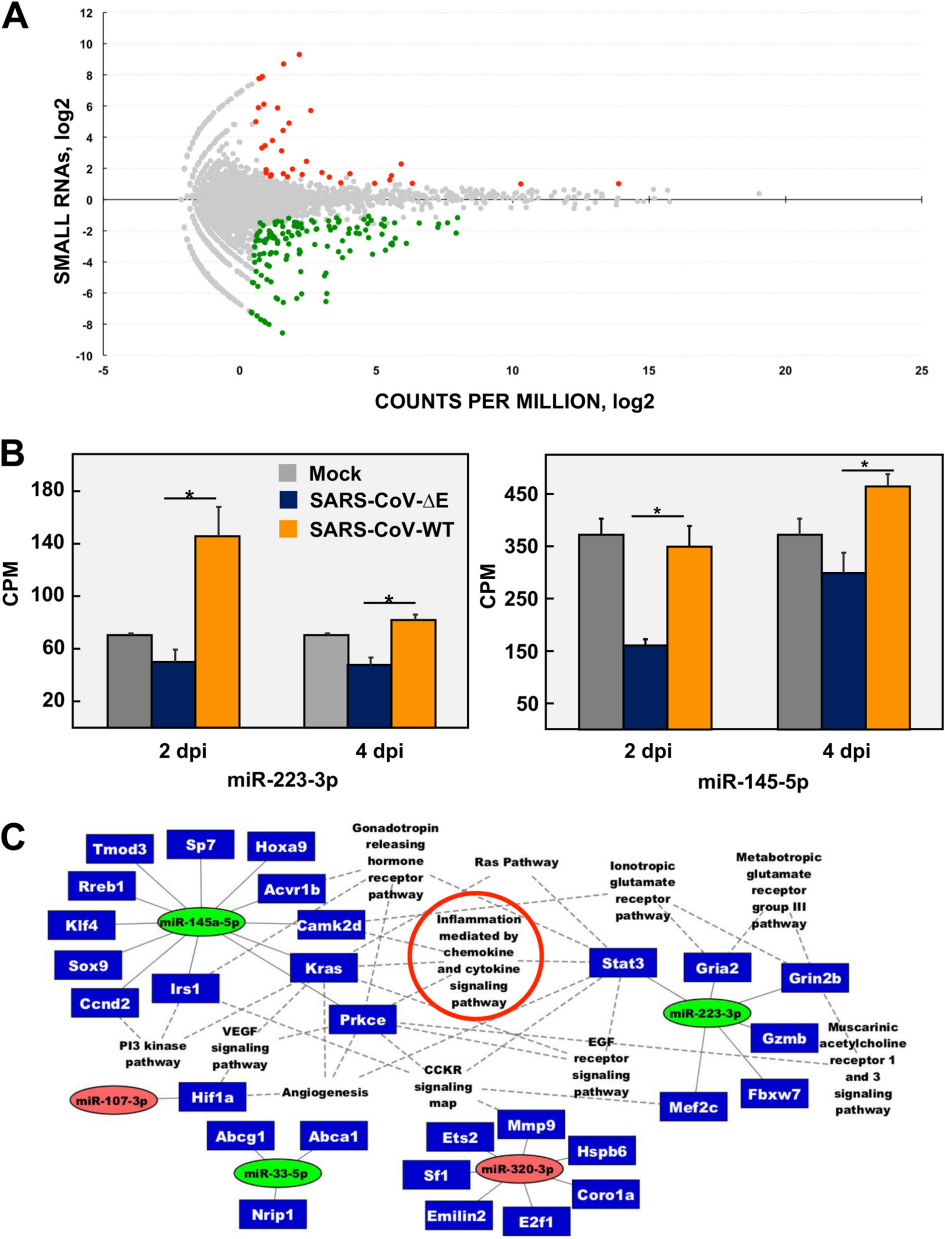
microRNAs differentially expressed in the lungs of mice infected with the attenuated SARS-CoV-ΔE compared to the virulent SARS-CoV-WT. (A) The log_2_ ratio of small RNA expression in SARS-CoV-ΔE to SARS-CoV-WT infection at 2 days p.i. is represented on the *y* axis. The log_2_ counts per million (CPM) for each small RNA sequence is represented on the *x* axis. Colored dots represent small RNAs that were differentially expressed with |fold change| ≥ 2; FDR ≤ 0.05; and average raw counts ≥ 20. Upregulated sequences are indicated in red and downregulated sequences in green. (B) CPM of miRNAs 223-3p and 145a-5p differentially expressed in SARS-CoV-ΔE compared to SARS-CoV-WT infection are represented; *, |fold change| ≥ 2 and FDR ≤ 0.05. (C) miRTarBase regulatory network of interactions between annotated mouse miRNAs differentially expressed in SARS-CoV-ΔE versus SARS-CoV-WT infection and mRNA targets validated with strong experimental evidence. The functional groups with *P* value < 0.1 according to the Panther Classification System are shown; miRNAs are represented in colored ovals according to their differential expression in SARS-CoV-ΔE versus WT infection. Green ovals indicate upregulated miRNAs (fold change ≤ −2), while red ovals indicate downregulated miRNAs (fold change ≥ 2). Target genes are shown inside blue rectangles. Continuous lines represent miRNA–mRNA interactions and dotted lines indicate gene-pathway interactions. Small RNAseq results were obtained from *n* = 3 mice per experimental condition (i.e., infected with each virus or mock-infected and sacrificed at 2 or 4 days p.i.). Statistical significance was calculated by two-tailed Student´s *t* test. *, *P* value < 0.05.

To address the relevance of miRNAs in SARS-CoV pathogenesis, BALB/c mice were infected either with virulent SARS-CoV-WT or attenuated mouse-adapted SARS-CoV-ΔE (8). Despite that both viruses only differ in the expression of E protein, they cause a significantly different lung pathology. SARS-CoV-WT causes a severe lung inflammation with abundant neutrophil infiltrates, alveolar edema, and high levels of pro-inflammatory cytokines that lead to the death of 100% infected mice. In contrast, SARS-CoV-ΔE infection leads to a mild lung pathology that does not cause any mortality in mice (8). Small RNAs (<200 nt) from the lungs of mice infected with SARS-CoV-ΔE or -WT at 2 and 4 dpi were analyzed by small RNAseq to determine differentially expressed (DE) cellular small RNAs (fold change [FC] > 2 or FC < −2), with statistical significance (false discovery rate [FDR] < 0.05). One hundred sixty-seven small cellular RNAs were differentially expressed in SARS-CoV-ΔE versus -WT infection at 2 dpi (Fig. 1A). Most of them (132 out of 167) were downregulated in infection with attenuated SARS-CoV-ΔE virus (Fig. 1A, green dots), while only a small number of RNAs (35 out of 167) was upregulated (Fig. 1A, red dots), suggesting that small RNAs contribute to host response to a virulent SARS-CoV infection. Only 23 out of 167 DE small RNAs were mouse annotated miRNAs (miRBase v.20 (36) (Table 1), indicating that other small cellular RNAs, or even novel nonannotated miRNAs, might be participating in the response to virus infection, as described for other viral respiratory infections (31). At 4 dpi, only 2 annotated miRNAs not related to potential antiviral responses, out of 59 small cellular RNAs, were differentially expressed. Therefore, further analysis was performed with DE miRNAs identified at 2 dpi. Differential expression of most annotated miRNAs in infection with virulent SARS-CoV-WT versus attenuated SARS-CoV-ΔE ranged from 2- to 4-fold (Table 1 and Fig. 1B). Similar moderate changes have been described for other miRNAs, albeit with a significant biological impact in pathogenesis (37, 38).

**TABLE 1:** Quantification by RNAseq of annotated miRNAs differentially expressed in SARS-CoV-ΔE versus SARS-CoV-WT infected mouse lungs^*a*^

		CPM	
miRNA	Fold-change	ΔE	WT
mmu-miR-6978-5p	−14.39	0.434	6.255
mmu-miR-331-3p	−3.27	5.333	17.448
mmu-miR-5099	−2.99	21.112	63.119
mmu-miR-33-5p	−2.98	90.824	269.660
mmu-miR-223-3p	−2.85	56.689	161.456
mmu-miR-296-5p	−2.64	1.613	4.257
mmu-miR-223-5p	−2.43	15.032	36.504
mmu-miR-3102-3p	−2.40	25.457	60.969
mmu-miR-145a-5p	−2.23	168.897	377.413
mmu-miR-107-3p	2.01	1782.888	891.444
mmu-miR-320-3p	2.02	21395.207	10623.710
mmu-miR-150-3p	2.05	115.360	56.103
mmu-miR-30c-1-3p	2.06	44.017	21.407
mmu-miR-760-3p	2.11	19.027	9.000
mmu-miR-351-3p	2.39	70.035	29.243
mmu-miR-3474	2.72	16.111	5.938
mmu-miR-5107-5p	2.73	5.560	2.035
mmu-miR-877-5p	2.88	80.171	27.761
mmu-miR-378d	3.17	29.243	9.254
mmu-miR-128-3p	3.29	3.555	1.079
mmu-miR-3572-5p	3.31	14.672	4.423
mmu-miR-483-5p	3.88	7.516	1.932
mmu-miR-184-3p	4.86	133.436	27.474

a Annotated miRNAs with FC > 2 or < −2, FDR < 0.05, and a number of raw counts > 20 in average are shown.

To investigate the functional impact of DE miRNAs, experimentally validated targets of 23 annotated miRNAs were determined using miRTarBase v.6.0 (39). Twenty-nine predicted mRNA targets were found for 5 miRNAs (miRNA-223-3p, 145a-5p, 33-5p, 107-3p, and 320-3p) (Fig. 1C). miRNAs-223-3p, 145a-5p and 33-5p were upregulated in SARS-CoV-WT versus ΔE-infected mouse lungs, while miRNAs-107-3p and 320-3p were significantly downregulated in WT vs ΔE infection, according to small RNAseq results (Table 1). These 5 miRNAs and their associated mRNA targets were subjected to functional enrichment analysis using Panther (http://pantherdb.org) (40) to show statistically significant pathways in which DE miRNAs were involved. Predicted miRNA–mRNA interactions and their functional relationship were represented as a network (Fig. 1C), illustrating that miRNAs may be part of a complex regulatory system in SARS-CoV lung infection. miR-223-3p and miR-145a-5p might regulate 6 and 12 targets, respectively, mainly involved in inflammation mediated by chemokine and cytokine signaling pathways, which are major contributors of severe inflammation associated with SARS-CoV-WT infection (6, 12, 41–44). These results suggested that miRNAs differentially expressed in the presence of viral virulence factor within E protein might be contributing to the regulation of host immunopathological response to SARS-CoV-WT infection. To evaluate this hypothesis, an integrative analysis of miRNAs and predicted target-mRNA expression changes was performed using transcriptome sequencing of long RNAs (>200 nt) from the same SARS-CoV-infected lung samples (Table 2). Only 3 (*IRS1*, *MEF2c*, and *GZMB*) out of 29 miRNA predicted mRNA targets showed statistically significant expression changes (FC > 2; FDR < 0.05) in SARS-CoV-WT versus ΔE infection. Moreover, the upregulation of *IRS1* and *MEF2c* mRNAs in SARS-CoV-ΔE versus WT infection correlated with miRNA-145a and 223 downregulation, respectively. These opposite changes in the expression of predicted miRNA-145a-*IRS1* and miRNA-223-*MEF2c* pairs (Fig. 1C) suggested a potential regulatory effect of miRNAs 145a and 223 (23).

**TABLE 2:** Quantification by RNAseq of differential expression of validated mRNA targets of differentially expressed miRNAs^*a*^

mRNA	FC	FDR	DESCRIPTION
IRS1	2.77	0.0003	Insulin Receptor Substrate 1
GZMB	−6.27	0.000	Granzyme B
MEF2C	2.58	0.001	Myocyte Enhancer Factor 2C

a Lung expression levels, quantified by RNA-seq, of validated mRNA targets (Fig. 1C) of miRNAs 223-3p, 145a-5p, 33-5p, 107-3p, and 320-3p, differentially expressed in the lungs of mice infected with SARS-CoV-ΔE or SARS-CoV-WT at 2 days p.i. Only mRNAs with FC > 2 or < −2 and FDR < 0.05 are shown.

### Validation of differences in expression of miR-223-3p and miR-145a-5p in SARS-CoV-infected cell cultures.

**FIG 2:**
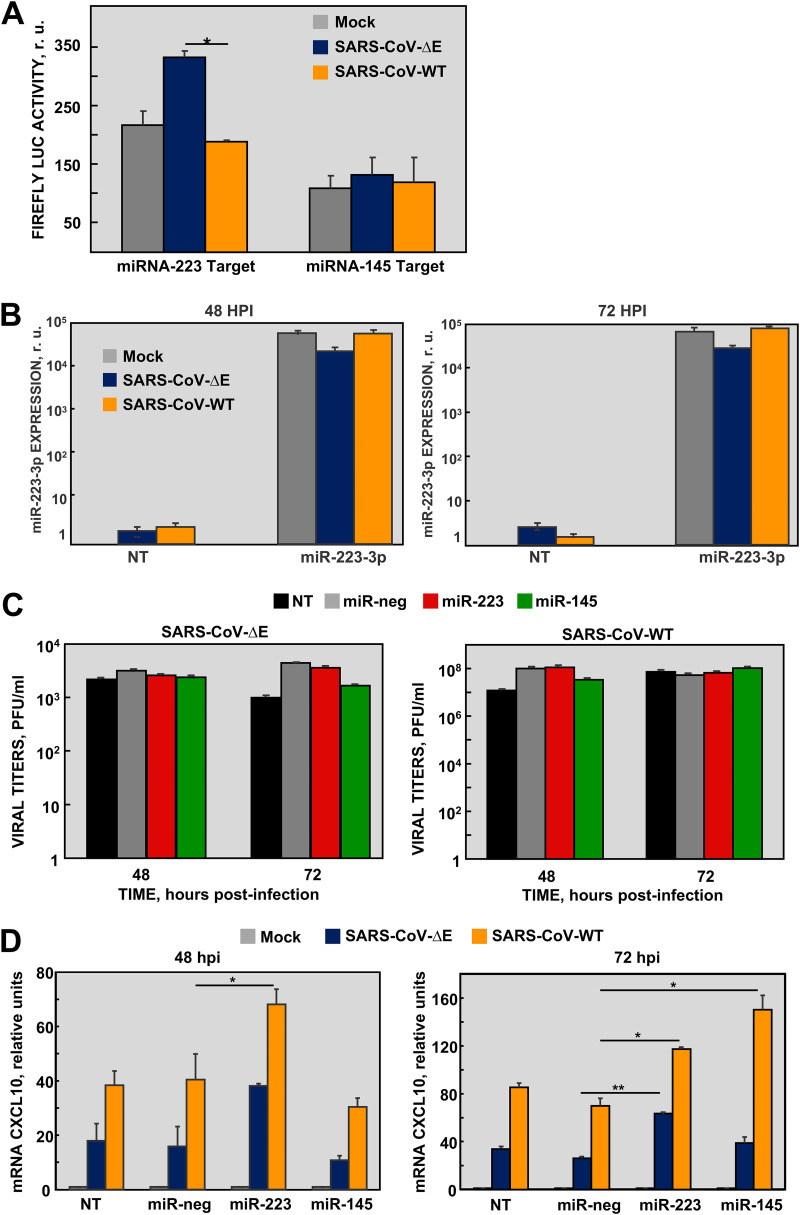
Effect of miRNAs in SARS-CoV-infected Calu-3 2B4 cells. (A) Calu-3 2B4 cells were transfected with luciferase reporter vectors containing the complementary targets for miR-223-3p or miR-145a-5p in the 3′ UTR. After 6 h, cells were infected with SARS-CoV-ΔE or -WT viruses. Luciferase activity was measured 48 h p.i. and normalized to the transfection efficiency, measured by RFP fluorescence. (B) Quantification by RT-qPCR of transfected miRNA-223-3p. Calu-3 2B4 cells were either nontransfected (NT) or transfected with RNA mimics (25 nM) of miR-223-3p 6 h prior to infection with SARS-CoV-ΔE or -WT at MOI 1. The levels of miR-223-3p into the cells at 48 and 72 h p.i. were referred to those in nontransfected and noninfected cells, using the ΔΔCt method and U6 snRNA as the normalization endogenous gene. (C) Viral titers in cells transfected with RNA mimics of miR-223-3p, miR-145a-5p, or miR-neg used as a negative control and infected with SARS-CoV-ΔE or -WT were measured from supernatants at the indicated times postinfection. (D) Quantification by qPCR of CXCL10 mRNA levels relative to those in mock-infected cells, using the ΔΔCt method and the 18s rRNA as the normalization endogenous control. Error bars indicate the standard deviation. Experiments were repeated at least twice with *n* = 3 biological replicates per experimental condition. Statistical significance was calculated by two-tailed Student´s *t* test. *, *P* value < 0.05; **, *P* value < 0.01.

Since miR-223-3p and miR-145a-5p have been extensively involved in inflammation processes, they were selected for further analysis (24, 45) (Fig. 1C). To validate the RNAseq differential expression of both miRNAs, two cell lines susceptible to SARS-CoV infection, mouse DBT-mACE2 and human lung Calu-3 2B4, were infected either with SARS-CoV-ΔE or -WT (MOI 1), and levels of miR-223-3p and miR-145a-5p were quantified by qPCR at different times postinfection (p.i.). In both cell lines, changes in miRNA levels were mostly below the sensitivity limit of qPCR (2-fold) (Fig. S1 in the supplemental material), which hindered the validation of small RNAseq results. Moreover, these results confirmed the limitations of using cell cultures to study *in vivo* function of cell miRNAs, since the whole lung contains different cells types, including infiltrating immune cells, which contribute to the expression of miRNAs. In fact, miRNA-223 is modestly expressed by human airway epithelial cells (46) and most miRNA-223 is produced by neutrophils and transferred to pulmonary cells by exocytosis (37). In order to increase miRNA detection sensitivity, a luciferase (LUC) reporter gene with miRNA target sequences at the 3′ UTR was used to analyze the post-transcriptional gene silencing (PTGS) effect of miRNAs. An increase in the expression of a specific miRNA in infected cells could be measured as a decrease in the LUC activity. LUC activity regulated by miR-223-3p increased 1.76-fold in lung Calu-3 2B4 cells infected with SARS-CoV-ΔE compared to WT at 48 h p.i. (Fig. 2A), suggesting that miR-223-3p was downregulated in SARS-CoV-ΔE infection in these cells, as observed *in vivo* (Fig. 1B). No significant changes were observed in LUC activity regulated by miR-145a-5p (Fig. 2A), indicating that this miRNA was not differentially expressed in these lung cells during SARS-CoV infection.

### Effect of miR-223-3p and miR-145a-5p mimics on inflammatory pathways during SARS-CoV infection in Calu-3 2B4 cells.

The effect of miR-223-3p and miR-145a-5p in the regulation of SARS-CoV induced inflammatory pathways was assessed in Calu-3 2B4 cells transfected with miRNA mimics. The efficient transfection of miRNA mimics into Calu-3 2B4 cells subsequently infected with SARS-CoV was confirmed by qPCR, showing stable levels of miR-223-3p at 48 and 72 h p.i., around 10^5^-fold higher than in the negative control-transfected (NT) cells (Fig. 2B). Transfection of miR-223-3p and miR-145a-5p mimics did not have a significant impact on SARS-CoV-WT and -ΔE titers as compared to nontransfected (NT) or negative miRNA (miR-neg)-transfected cells used as controls (Fig. 2C). The expression of *CXCL10* mRNA, which is one of the most upregulated pro-inflammatory cytokines in lungs of mice infected with virulent SARS-CoV-WT versus SARS-CoV-WT-ΔE (12), was analyzed by qPCR. As expected, in the absence of miRNA mimics (NT and miR-neg controls), *CXCL10* levels were increased in cells infected with virulent SARS-CoV-WT as compared to ΔE virus, both at 48 and 72 h p.i. (Fig. 2D). Transfection of miRNA-223-3p mimics significantly upregulated *CXCL10* in Calu-3 2B4 cells infected with SARS-CoV-WT (at 48 and 72 h p.i) or SARS-CoV-ΔE (at 72 h p.i), suggesting that in this cell line, miRNA-223-3p regulated the pro-inflammatory response, mainly at late times postinfection. Transfection of miR-145a-5p mimics only induced the expression of *CXCL10* in SARS-CoV-WT infected cells at 72 h p.i. Since miRNA-223-3p provided more consistent results in terms of activation of the pro-inflammatory response, it was selected for further *in vivo* experiments.

### Effect of miR-223-3p inhibition in SARS-CoV infected mice.

**FIG 3:**
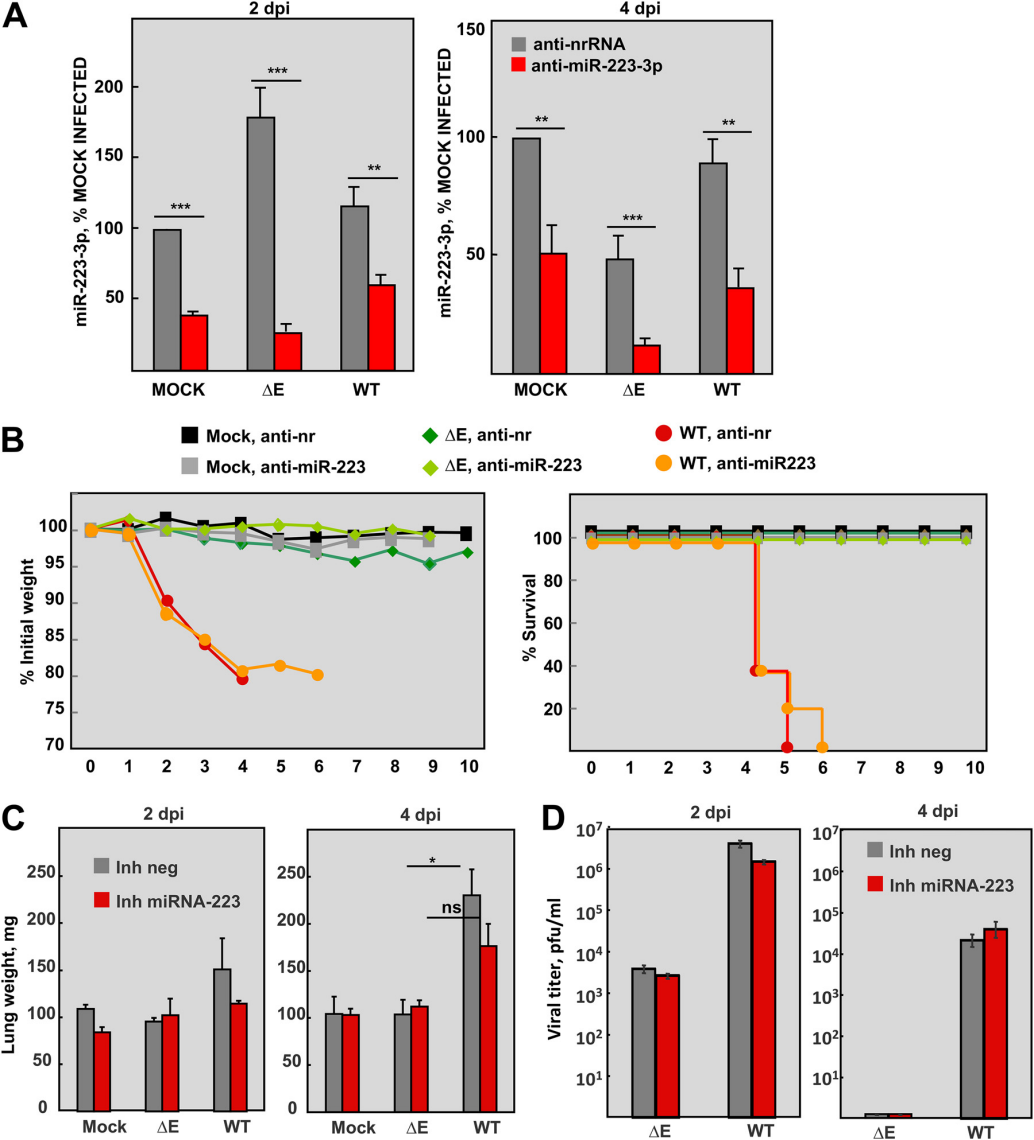
Analysis of miRNA-223-3p in SARS-CoV-infected mice using antisense LNA inhibitors. BALB/c mice were intranasally inoculated with 200 μg (10 mg/kg) of the LNA miRNA-223-3p inhibitor or a nonrelated sequence (nrRNA) as a negative control. After 24 h, the mice were infected with 10^5^ PFU of SARS-CoV-ΔE or -WT and their lungs were collected at 2 and 4 days p.i. *n* = 11 mice were mock-infected or infected with each virus and treated with miRNA-223-3p inhibitor or a negative control. At 2 and 4 days p.i., *n* = 3 mice per experimental condition were sacrificed for analysis of lung RNA, viral titer, and histopathology. (A) RT-qPCR analysis of miRNA-223-3p levels in the lungs, relative to the expression in mock-infected mice inoculated with the nrRNA. The ΔΔCt method was used for relative quantification, with snRNA-U6 as the endogenous control. (B) The weight loss (left graph) and survival (right graph) of mice was monitored during 10 days. Weight loss was expressed as the percentage of the initial weight measured before infection. (C) Weight of the lungs of mice at 2 and 4 days p.i. Lungs were weighted when collected and prior to fixation. (D) Viral titers in the lungs of infected mice. At 2 and 4 days p.i., 3 mice from each group were sacrificed to determine virus titers. Error bars indicate the standard error of the mean from 3 mice lungs per each condition. Statistical significance was calculated by two-tailed Student´s *t* test. ns, nonsignificant; *, *P* value < 0.05.

*In vivo* systems provide a more physiological context for miRNA studies, since miRNA expression is cell type-specific and its biological effect depends on the cellular milieu of target mRNAs in each environmental condition (47). The relevance of miRNA-223-3p in lung inflammation induced by SARS-CoV was assessed by miRNA-inhibition assays in infected mice. BALB/c mice were intranasally administered with a single dose (10 mg/kg) of LNAs anti-miR-223-3p or a nonrelated negative control (anti-nr-RNA) 24 h prior to infection with either SARS-CoV-ΔE or -WT. LNAs used *in vivo* included chemical modifications to increase stability and inhibitory efficacy and to minimize off-target and immunostimulatory effects. Relative changes in lung miRNA-223-3p levels after inhibition were confirmed by RT-qPCR at 2 and 4 days p.i. (Fig. 3A). At 2 days p.i., when miR-223-3p peaked in SARS-CoV-infected lungs (Fig. 1B), levels of miR-223-3p were significantly reduced by the specific inhibitor compared to nrRNA inhibitor (60%, 85%, and 49% reduction in mock-infected, SARS-CoV-ΔE, and SARS-CoV-WT infected mice, respectively). The highest inhibition efficiency was observed in SARS-CoV-ΔE infection, at both 2 and 4 days p.i., suggesting that lung environment in this attenuated infection might favor the inhibitory action of anti-miR-223-3p LNAs. RT-qPCR is an appropriate technique to quantify the inhibitory effect of antisense RNAs (Fig. 3A). However, RNAseq provides higher accuracy and sensitivity to identify changes in expression of moderately abundant miRNAs during infection by SARS-CoV mutants (Fig. 1B).

During the course of the experiment, treatment of mock-infected mice with miR-223-3p inhibitor did not induce any adverse effects such as weight loss, movement difficulties, lethargy, or unhealthy appearance, as compared to mice treated with negative control inhibitor. Mice infected with attenuated SARS-CoV-ΔE and treated with anti-nrRNA did not significantly lose weight, compared to mock-infected animals, and all of them survived at 10 days p.i. (Fig. 3B). Inhibition of miR-223-3p in SARS-CoV-ΔE infection led to a sustained, although not significant, increase in the weight of mice compared to those treated with the anti-nrRNA (Fig. 3B). In contrast, mice infected with virulent SARS-CoV-WT rapidly lost weight (Fig. 3B) and died, although a 1-day delay in death was observed in the group treated with anti-miR-223-3p inhibitor compared to those treated with anti-nrRNA (Fig. 3B). Although these differences were not statistically significant, in part because of the high variability and small sample size of *in vivo* experiments, inhibition of miR-223-3p in SARS-CoV-ΔE and SARS-CoV-WT infected mice was associated with a trend toward improved outcome of infection, suggesting that miRNA-223-3p might be contributing to SARS-CoV-induced morbidity and mortality.

### Effect of miR-223-3p inhibition in SARS-CoV lung pathology.

Lung edema induced by SARS-CoV-WT correlates with a significant increase in lung weight (13, 48). As expected, the weight of SARS-CoV-WT-infected lungs treated with the negative control significantly increased at 4 days p.i., compared to SARS-CoV-ΔE infection (*P* = 0.0167) (Fig. 3C). In contrast, treatment with anti-miRNA-223-3p inhibitor at 4 days p.i. diminished the weight increase of SARS-CoV-WT-infected lungs compared to SARS-CoV-ΔE infection, leading to a nonsignificant difference (*P* = 0.0571). These results suggested that miRNA-223-3p was contributing to SARS-CoV-WT-induced pulmonary edema. Inhibition of miRNA-223-3p did not significantly affect titers of SARS-CoV-WT and SARS-CoV-ΔE in lungs of infected mice (Fig. 3D), suggesting that the smaller increase of lung weight in WT versus ΔE infection at 4 days p.i. was not caused by lower viral growth, but by the host inflammatory response to infection.

**FIG 4:**
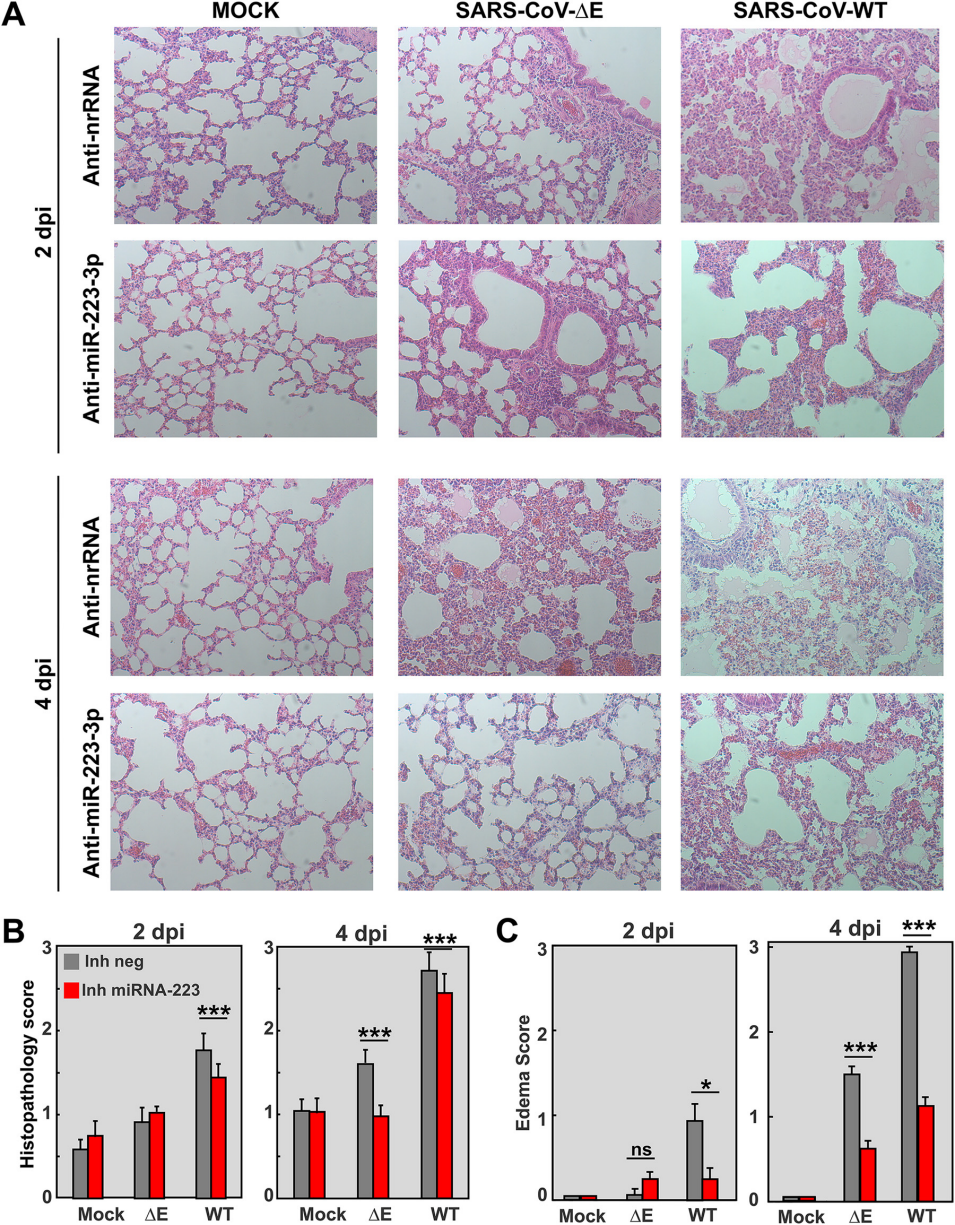
Effect of inhibition of miRNA-223-3p in the lung histopathology of SARS-CoV-infected mice. (A) Histopathological analysis of lungs of mice treated with miRNA-223-3p antisense LNAs and infected with SARS-CoV-ΔE and WT viruses, as described in Fig. 3. Tissue sections were prepared from lungs at 2 and 4 days p.i. and stained with hematoxylin and eosin. Three independent mice per group were analyzed. Images are representative of 50 microscopy fields observed for each independent mouse. (B and C) Scoring of lung histopathology at 2 and 4 days p.i. Pathology of mouse lungs was scored in a blinded fashion using 3 mice per condition according to the presence of inflammatory cell infiltrates (B) and edema in air spaces (C), as described in Materials and Methods. A scale of 0 (none) to 3 (severe) was used, according to previously described procedures (49, 50). Mean values are represented. Error bars indicate standard error of the mean. Statistical significance was calculated by two-tailed Student´s *t* test. ns, nonsignificant; *, *P* value < 0.05; ***, *P* value < 0.001.

Pulmonary histopathology in SARS-CoV-infected mice treated with anti-miRNA-223-3p inhibitor (Fig. 4A) was scored from lung sections at 2 and 4 days p.i., according to the presence of interstitial, peribronchiolar, and perivascular cell infiltrates (49) (Fig. 4B) and edema in air spaces (Fig. 4C) (50). As expected, in anti-nrRNA-treated mice, infection with virulent SARS-CoV-WT led to significant cell infiltration and edema accumulation in alveolar and bronchiolar spaces, more pronounced at 4 days p.i., compared to infection with attenuated SARS-CoV-ΔE. In contrast, treatment with anti-miRNA-223-3p significantly reduced inflammation signs, mainly edema, both in SARS-CoV-WT and ΔE infection at 2 and 4 days p.i. (Fig. 4C), supporting that miRNA-223-3p was contributing to lung edema induced by SARS-CoV.

### Effect of miR-223-3p inhibition in inflammatory cytokines induced by SARS-CoV in lungs of infected mice.

**FIG 5:**
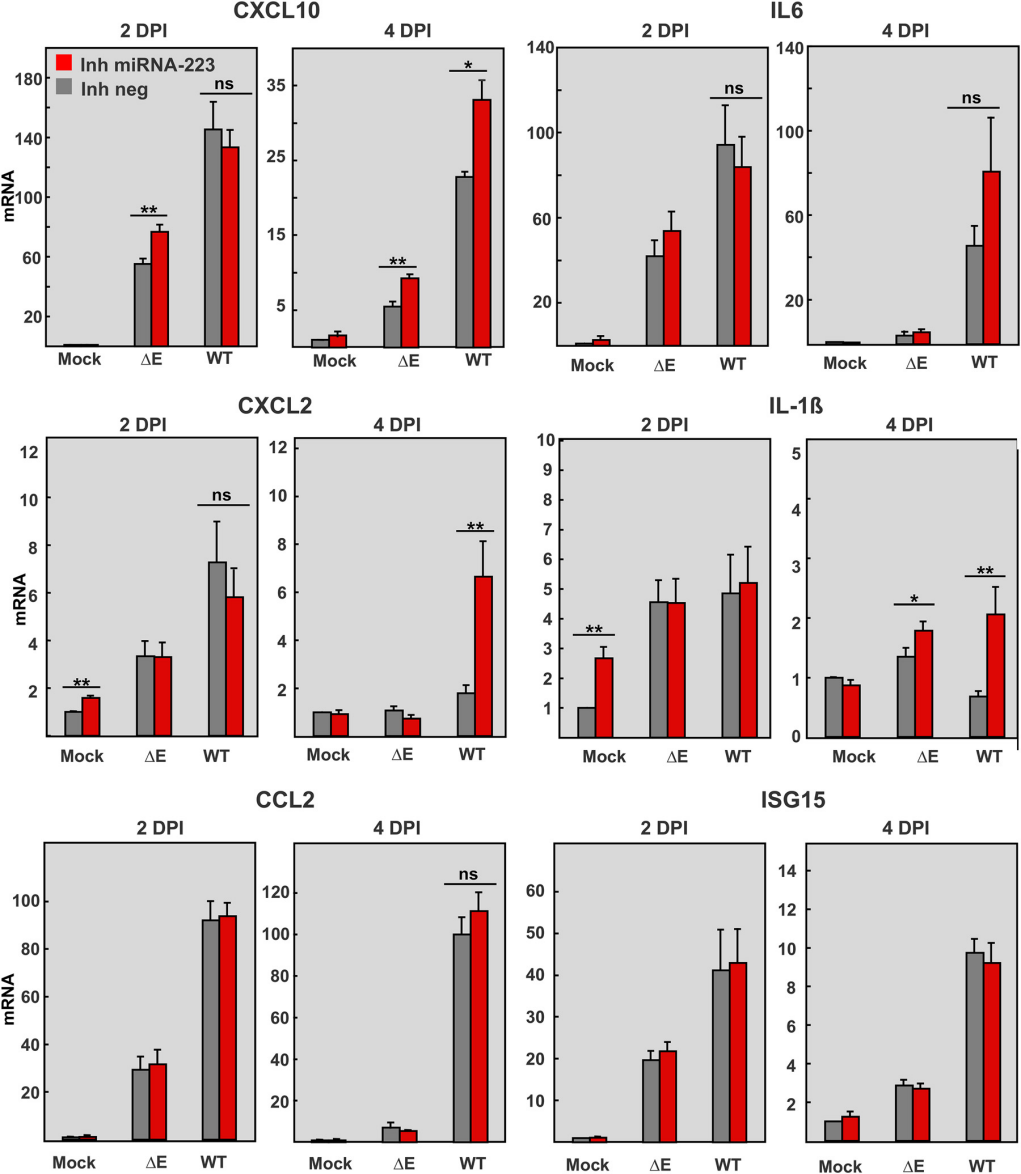
Effect of miRNA-223-3p inhibition in the expression of pro-inflammatory cytokines and chemokines in SARS-CoV-infected mice. mRNA quantification by RT-qPCR of the indicated inflammatory mediators at 2 and 4 days p.i. in the lungs of mice treated with miRNA-223-3p antisense LNAs and infected with SARS-CoV-ΔE and WT viruses, as described in Fig. 3. mRNA levels were made relative to those in mice treated with a negative control LNA (Inh neg) and mock-infected, using the ΔΔCt method and the 18s rRNA as the normalization endogenous control. Error bars indicate the standard error. Statistical significance was calculated by two-tailed Student´s *t* test. ns, nonsignificant; *, *P* value < 0.05; **, *P* value < 0.01.

To evaluate the potential of miR-223-3p to modulate the inflammatory response during SARS-CoV infection, mRNA expression levels of relevant proinflammatory cytokines (IL-6, IL-1ß) and chemokines (CCL2, CXCL10, and CXCL2) were analyzed by qPCR (Fig. 5). As expected, SARS-CoV-WT infection induced at 2 days p.i. in the lungs a significantly higher expression of proinflammatory cytokines (*CXCL10*, *CXCL2. CCL2*, and *IL-6*) than SARS-CoV-ΔE (Fig. 5). Inhibition of miR-223-3p did not impact levels of CCL2, IFN-stimulated gene 15 (*ISG15*), or IL-6 in either SARS-CoV-WT or ΔE infection at any time point. In contrast, expression in the lungs of *CXCL10*, *CXCL2*, and *IL-1ß* increased after inhibition of miR-223-3p, mainly at 4 days p.i., suggesting that the regulatory effect of miR-223-3p might be dynamic and dependent on the tissue inflammatory environment. In a context of lower levels of pro-inflammatory cytokines, as that induced by SARS-CoV-ΔE or SARS-CoV-WT at 4 days p.i., miRNA-223-3p might have an anti-inflammatory effect by repressing the expression of *CXCL10*, *CXCL2*, and IL-1ß (51). However, in a context of “cytokine storm,” as that induced by SARS-CoV-WT at 2 days p.i. (13, 48), fine-tuning regulation of inflammation by miRNA-223-3p would not be significant.

### Effect of miR-223-3p inhibition in other miRNA targets in lungs of infected mice.

**FIG 6:**
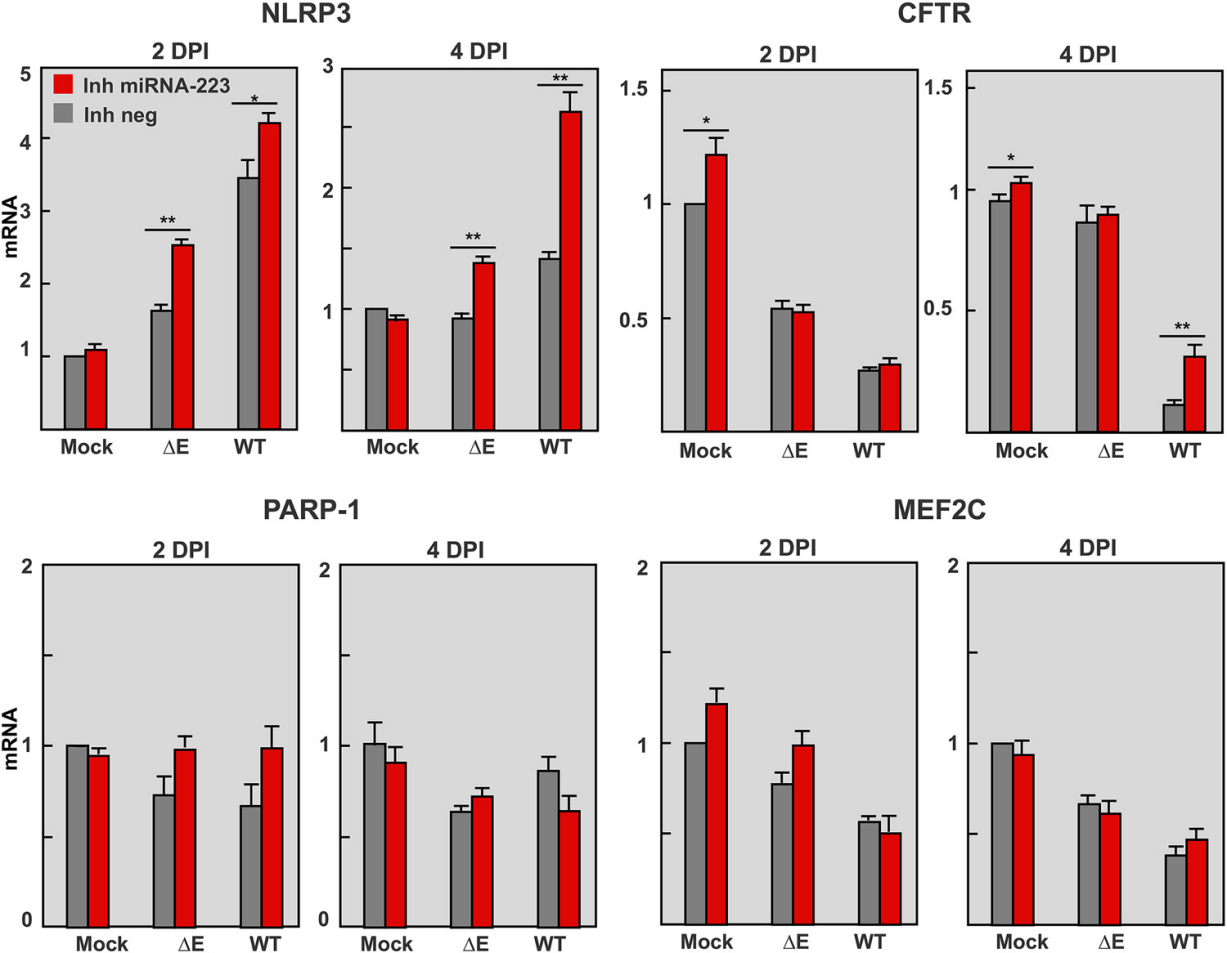
Effect of miRNA-223-3p inhibition in the expression of validated targets of miRNA-223-3p in the lungs of SARS-CoV-infected mice. Quantification of IRS1, HEY1, NLRP3, and MEF2C mRNAs, which are validated targets of miRNA-223-3p (56), at 2 and 4 days p.i. RT-qPCR of RNA from the lungs of mice treated with miRNA-223-3p antisense LNAs and infected with SARS-CoV-ΔE and WT viruses was performed as described in Fig. 5. The ratio of mRNA levels in infected to mock-infected lungs is represented, as calculated by the ΔΔCt method using 18S rRNA as an endogenous control. Error bars indicate the standard error. Statistical significance was calculated by two-tailed Student´s *t* test. *, *P* value < 0.05; **, *P* value < 0.01.

E protein ion channel activity contributes to SARS-CoV-WT-induced inflammation through the activation of NLRP3 inflammasome (14). Since miRNA 223 was described to target *NLRP3* mRNA in intestinal inflammation (38, 52), the effect of miRNA-223-3p on lung *NLRP3* mRNA levels was analyzed in SARS-CoV infected mice. Inhibition of miRNA-223-3p led to a significant increase in *NLRP3* mRNA at 2 and 4 days p.i., thus confirming that *NLRP3* was a target of miRNA-223-3p during SARS-CoV infection (Fig. 6). Accordingly, NLRP3 dependent cytokines such as *IL-1ß* and *CXCL10* (38) were also increased (Fig. 5). These results suggested that downregulation of NLRP3 inflammasome by miR-223-3p might provide an additional negative feedback mechanism of lung inflammation. Most validated miRNA-223-3p targets (*NLRP3*, *CXCL2*, *IL-6*, etc.) (51) are pro-inflammatory factors, and their increase in the lungs of mice after miRNA-223-3p inhibition should be contributing to an enhanced inflammation. The significant reduction in pulmonary inflammation observed in SARS-CoV infected mice (Fig. 4) suggested that other anti-inflammatory cell targets would also be increased by miRNA-223-3p inhibition, as expected according to the pleiotropic nature of miRNAs.

**FIG 7:**
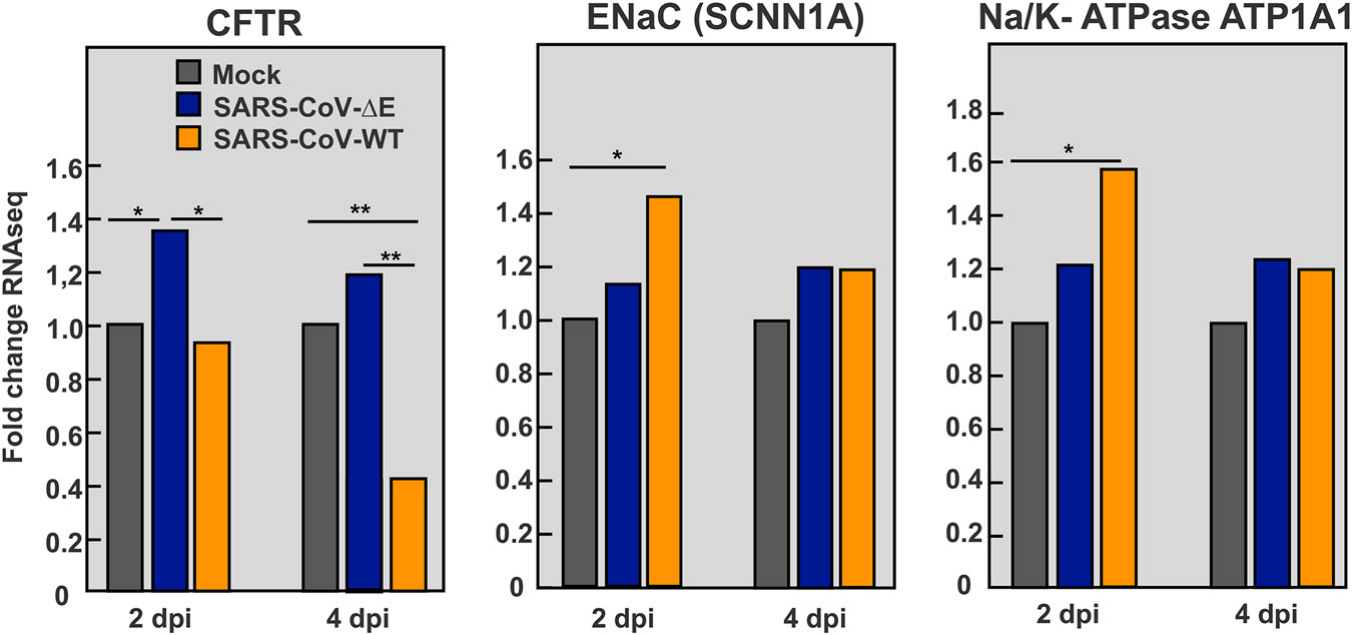
Differential expression of ion transporter mRNAs in the lungs of SARS-CoV-infected mice. RNAseq quantification of mRNAs from the lungs of mice infected with SARS-CoV-ΔE and WT viruses at 2 and 4 days p.i. Statistical significance was calculated by two-tailed Student´s *t* test. *, *P* value < 0.05; **, *P* value < 0.01.

*CFTR* is a miRNA-223-3p target (53) involved in the resolution of pulmonary edema (33, 54). *CFTR* mRNA levels were significantly reduced in the lungs of SARS-CoV-WT versus ΔE-infected mice, as confirmed by qPCR (Fig. 6) and RNAseq (Fig. 7). This effect was particularly observed at 4 days p.i., when *CFTR* expression was less than 15% of that in mock-infected and ΔE-infected lungs (Fig. 6), in agreement with a more severe inflammatory pathology in WT infection. miRNA-223-3p inhibition led to a significant increase in *CFTR* mRNA in SARS-CoV-WT infected lungs at 4 days p.i. (Fig. 6), supporting that *CFTR* was contributing to reduction in pulmonary edema (Fig. 4). In contrast to downregulation of *CFTR* during SARS-CoV-WT infection, no reduction was observed in RNAseq expression of other ion transporters also involved in fluid clearance from airspaces, such as epithelial sodium channel (*ENaC*) and Na/K-ATPase (Fig. 7) (55).

No significant differences in mRNA levels of other validated miRNA-223-3p targets with pro-inflammatory activity, such as *MEF2C* and *PARP-1* (56) (Fig. 6), were observed, supporting that miRNA-223-3p regulates specific targets in the context of SARS-CoV infection.

## DISCUSSION

In this article, we study the contribution of host miRNAs to severe lung inflammatory pathology induced by SARS-CoV E protein. Small RNAseq analysis of SARS-CoV infected mouse lungs identified miRNA-223 as a potential regulator of pulmonary inflammation, since it was significantly increased in virulent SARS-CoV-WT versus attenuated SARS-CoV-ΔE infection. Although SARS-CoV-ΔE grows to lower titers than SARS-CoV-WT, mutations of different virulence motifs in E protein attenuated the virus without affecting viral growth, demonstrating that E protein is a viral virulence factor (13, 14). *In vivo* inhibition of miRNA-223-3p increased the expression of different host factors involved in the regulation of inflammatory response and also reduced pulmonary inflammation, suggesting that miRNA-223 participates in the complex and dynamic regulation of inflammatory response induced by SARS-CoV E protein. In particular, miRNA-223-3p inhibition increased mRNA levels of validated targets *CXCL2* (51), *CFTR* (53, 56), *NLRP3*, and NLRP3-dependent proinflammatory cytokines *IL-1ß* and CXCL10 (57–60). These results suggested that during infection, miRNA-223 contributes to restrict NLRP3 inflammasome activation and cytokine-mediated inflammation, but also to downregulate the epithelial anion transporter *CFTR*, involved in edema resolution (33, 54, 61). Interestingly, miRNA-223 inhibition in mice significantly reduced lung edema, which is also a common pathological finding in SARS-CoV and SARS-CoV-2 infection in humans. Therefore, miRNA-223 represents a potential therapeutic target in viral infections that cause severe lung inflammation, such as those induced by SARS-CoV and SARS-CoV-2.

miRNAs are induced by inflammatory stimuli and contribute to the regulation of innate immunity and inflammation. Different miRNAs work coordinately to produce a regulatory tension that evolves over time in the inflammatory milieu (62) in order to provide an efficient and fast response, which needs to be self-limited to prevent the pathological consequences of dysregulation (63, 64). The relevance of host miRNAs to the response against viral infections is still under debate. Using primary cell models that mimic viral infection, miRNAs were associated with regulation of pro-inflammatory cytokines and chemokines following prolonged stimulation periods (>1 day) (30). In agreement with this observation, miRNA-223 showed here an effect on inflammation particularly at 4 dpi. Lung inflammatory pathology induced by SARS-CoV was more evident at 4 days p.i. (Fig. 4B and C), despite mRNA levels of pro-inflammatory cytokines (CXCL10, IL-6, CXCL2, IL-1ß) at this time point being reduced, compared to 2 days p.i. (Fig. 5) (12). Similarly, differential expression of miRNAs was more significant at 2 days p.i., suggesting that early transcriptional and post-transcriptional activation of the inflammatory response was enough to induce the inflammatory phenotype observed at 4 days p.i. (Fig. 4).

It has been reported that viruses that activated host antiviral signaling also inhibited RISC activity required for miRNA gene silencing in mammalian cells (65). However, SARS-CoV expresses several IFN antagonists that efficiently prevent activation of host antiviral effectors (66), and RISC activity was not inhibited during SARS-CoV infection (67).

A number of host miRNAs have been proposed to affect virus-induced pathogenesis, although further research on their mechanism of action is still required (68). miR‐223 regulates the differentiation of myeloid cells, including neutrophils, monocytes, and granulocytes, which are essential in innate immune response (69, 70). miR-223 can be transferred from neutrophils to epithelial cells through exosomes or high-density lipoproteins (37, 71, 72) as a mechanism to restrict the magnitude of inflammation (46), particularly acute lung inflammation induced by mechanical ventilation or *Staphylococus aureus* infection (37). In SARS-CoV infection, neutrophils contribute to the severity of lung inflammation, mainly through the release of pro-inflammatory cytokines (6). Lower numbers of infiltrating neutrophils were detected in the lungs of mice infected with attenuated SARS-CoV-ΔE versus virulent SARS-CoV-WT (12), in line with lower miRNA-223-3p levels observed in SARS-CoV-ΔE infection (Fig. 1B). Therefore, neutrophils might provide an additional mechanism to regulate the inflammatory response through the transfer of miRNA-223-3p to lung epithelium during SARS-CoV infection. miRNA-223 has been shown to limit a variety of inflammatory processes (52, 57, 73). During inflammatory intestinal disease, miRNA-223 elevation contributed to limit inflammation by constraining NLRP3 inflammasome (38). In fact, an increased amount of NLRP3 protein was detected *in vivo* in miR-223-deficient mice (59) in association with neutrophilia, inflammatory lung disease (74), and increased levels of *CXCL2* (51). miRNA-223 was suggested to contribute to influenza virus pathogenicity, since it was strongly upregulated in the lungs of mice infected with either the recombinant 1918 pandemic virus (75) or a virulent strain, while miRNA-223 inhibition reduced the mortality of infected mice (76). Our results support a silencing effect of miRNA-223 on pro-inflammatory targets *CXCL2* and *NLRP3*. However, *in vivo* inhibition of miRNA-223 in SARS-CoV-WT or ΔE infected mice was also associated with a decrease in lung histopathology, suggesting that other miRNA-223 targets should be contributing to the phenotype. In particular, mRNA levels of *CFTR* anion transport significantly increased with miRNA-223-3p inhibition, suggesting that *CFTR* mRNA was targeted by miRNA-223 in SARS-CoV infected lungs. *CFTR* inactivation is associated with defects in alveolar epithelial fluid transport and, as a consequence, in the resolution of alveolar edema (33, 35, 54, 61, 77, 78). A number of studies showed the direct or indirect regulatory impact of miRNAs on expression of *CFTR* mRNA (79, 80).

Influenza M2 membrane protein, which has ion channel activity, was shown to inhibit *CFTR* during infection (81). Similarly, respiratory syncytial virus inhibited *CFTR* in primary cell cultures and in animal models (82), suggesting that *CFTR* inhibition is a common mechanism of pathogenesis in respiratory viruses. Infection with virulent SARS-CoV-WT significantly reduced the expression of *CFTR* in lungs of mice, especially at 4 days p.i., overlapping with the most prominent pulmonary edema (Fig. 4). The partial rescue of CFTR expression by miRNA-223 inhibition was associated with a decrease in lung edema, indicating that CFTR might be contributing to the resolution of alveolar edema induced by SARS-CoV.

**FIG 8:**
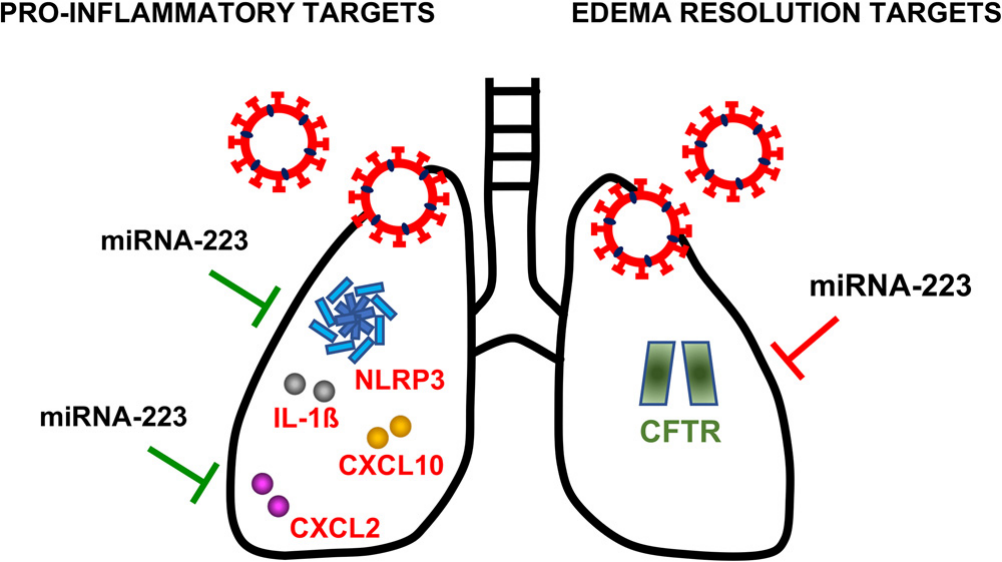
Overview of validated targets of miRNA-223 that could contribute to E protein-mediated pathogenesis in SARS-CoV infection. This scheme illustrates validated targets of miR-223 upregulated in mice treated with miRNA-223 antisense LNAs, which supports they are miRNA-223 targets during SARS-CoV infection. Red letters indicate mRNA targets with pro-inflammatory effects. Silencing of these mRNAs by miRNA-223 during infection would contribute to downregulate inflammation, as represented by green lines. CXCL2, Chemokine (C-X-C motif) ligand 2; NLRP3 inflammasome, NOD-, LRR-, and pyrin domain-containing protein 3; CXCL10, C-X-C motif chemokine ligand 10; IL-1ß, Interleukin 1 beta. Green letters indicate mRNA targets involved in edema resolution. Silencing of this mRNA by miRNA-223 during infection would contribute to edema formation, as represented by red lines. CFTR, cystic fibrosis transmembrane conductance regulator.

Overall, these results reflect the complex and dynamic network of mRNA–miRNA interactions occurring during SARS-CoV-induced inflammatory response. As a result of these pleiotropic effects, *in vivo* inhibition of miRNA-223-3p reduced pulmonary histopathology, suggesting that the impact of silencing anti-inflammatory targets, such as *CFTR*, was dominant over pro-inflammatory targets (Fig. 8), which are highly dysregulated in SARS-CoV infection.

Inflammatory responses modify miRNA biogenesis (64). Moreover, pro-inflammatory cytokines induced by viral infection have been associated with miRNA upregulation (83). Activation of innate defense pathways, such as toll-like receptor (TLR) signaling, results in miRNA induction, mostly dependent on NF-κB activity (28). Increased NF-KB activation and TNF-α production induced by E protein during SARS-CoV-WT infection (12) might be contributing to the observed miRNA-223 upregulation in WT versus SARS-CoV-ΔE-infected mice.

This is the first report on *in vivo* relevance of miRNA-223-3p in the regulation of inflammatory response mediated by E protein in SARS-CoV virulent infection. Partial inhibition of miRNA-223 by intranasal administration of antisense LNAs reduced SARS-CoV-induced lung edema, suggesting a therapeutic potential of miRNAs during SARS-CoV infection. The mature sequence of miR-223 is conserved within vertebrates (84), and its target sequence in mRNAs is also conserved across mammalian species (52), suggesting that regulatory effects of miRNA-223 observed in SARS-CoV mouse models might also be functional in humans during SARS-CoV and SARS-CoV-2 infection.

## MATERIALS AND METHODS

### Ethics statement.

Animal experimental protocols were approved by the Ethical Committee of the Center for Animal Health Research (CISA-INIA) (permit numbers: 2011–009 and 2011–09) and the DG Environment of Community of Madrid (PROEX 112/14) in strict accordance with Spanish National Royal Decree (RD 1201/2005) and international EU guidelines 2010/63/UE about protection of animals used for experimentation and other scientific purposes and Spanish Animal Welfare Act 32/2007. All work with infected animals was performed in a BSL3 laboratory of the Center for Animal Health Research (CISA-INIA).

### Mice.

Specific-pathogen-free 8-week-old BALB/c OlaHsd female mice were purchased from Harlan. Mice were maintained for 8 additional weeks in the animal facility at the National Center of Biotechnology (CNB-CSIC, Madrid). Sixteen-week-old mice were inoculated as described below with recombinant SARS-CoV-WT or SARS-CoV-ΔE in 50 μL of Dulbecco's modified Eagle's medium (DMEM) containing 2% fetal bovine serum (FBS, Biowhittaker). Mice were sacrificed at days 2 and 4 postinfection and lung samples were collected. For the miRNA-223-3p inhibition experiment, mice were monitored for 10 days for weight loss and survival. Animals reaching weight losses higher than 25% of the initial body weight were sacrificed according to the established euthanasia protocols. Infected mice were housed in a ventilated rack (Animal Transport Unit– Bio-Containment Unit, Allentown) in a BSL3 laboratory (CISA-INIA).

### Viruses.

The mouse-adapted (MA15) (85) recombinant SARS-CoV-WT and SARS-CoV-ΔE viruses were rescued from infectious cDNA clones generated in bacterial artificial chromosomes (BAC) in our laboratory (8). Virus titrations were performed in Vero E6 cells as described below.

### *In vivo* infections.

Sixteen-week-old female mice were intranasally inoculated with 10^5^ PFU of SARS- CoV-WT or SARS-CoV-ΔE. For the miRNA-223-3p inhibition experiment, 24 h prior to infection mice were intranasally inoculated with 200 mg (10 mg/kg) of the inhibitor (miRCURY LNA microRNA Inhibitor, Exiqon). For intranasal inoculations, mice were lightly anesthetized with isoflurane, and then 50 μL of solution containing either the miRNA inhibitor or the virus was laid on the nostrils of the mouse using a pipette tip. The small droplet was naturally inhaled by the mouse about 2–3 s later. Since this procedure was noninvasive and did not require a relevant physical intervention, it did not cause any inflammation in the mice.

### Lung samples from SARS-CoV-infected mice.

To analyze SARS-CoV titers, one-quarter of the right lung was homogenized in 2 mL of phosphate-buffered saline (PBS) containing 100 UI/mL penicillin, 100 mg/mL streptomycin, 50 mg/mL gentamicin, and 0.5 mg/mL fungizone using a MACS homogenizer (Miltenyi Biotec) according to manufacturer’s protocols. Virus titrations were performed as described below. To isolate long and small RNAs separately, one-half of the right lung was homogenized in 2 mL of Lysis/Binding Solution (mirVana miRNA isolation kit, Ambion) using a MACS homogenizer (Miltenyi Biotec), according to manufacturer’s protocols. Small and long RNAs were extracted separately from total RNA samples using mirVana miRNA Isolation protocol. To examine lung histopathology, the left lung of infected mice was fixed in 10% zinc formalin at 4° C for 24 h, and then it was embedded in paraffin. Serial longitudinal 5 μm sections were stained with hematoxylin and eosin by the Histology Service at the National Center of Biotechnology (CNB, Spain) and subjected to histopathological examination with a ZEISS Axiophot fluorescence microscope. Samples were obtained using a systematic uniform random procedure, consisting of serial parallel slices made at a constant thickness interval of 50 μm. Histopathology analysis was conducted in a blind manner by acquiring images of 50 random microscopy fields from around 40 nonadjacent sections for each of the three independent mice analyzed per treatment group. The measurement of inflammation damage was scored using a severity scale from 0 (absent) to 3 (severe with the presence of interstitial, peribronchiolar, and perivascular inflammation), as described (49). Tissues were also scored for the extent of edema in lung air spaces from 0, absence of edema; 1, edema detected in <33% of lung; 2, edema in 34–66% of lung; to 3, edema in >66% of lung (50).

### Deep sequencing of lung small and long RNAs.

Small and long RNAs were isolated (mirVana miRNA isolation kit, Ambion) from uninfected (*n* = 4 biological replicates) and SARS-CoV-ΔE or -WT infected mouse lungs (*n* = 3 replicates for each virus). The integrity of small and long RNAs was analyzed using the Bioanalyzer 2100 expert_Eukaryote. One μg of the small RNA fraction was used for library construction (BGI Genomics, Hong Kong) and subjected to 50SE sequencing on a Hiseq 2000 sequencer (Illumina), resulting in 20–40 million reads per sample. Six μg of the long RNA fraction was treated to eliminate rRNA (RiboZero rRNA Removal Kit, Epicentre), and libraries were constructed (NEBNext Ultra directional Library prep A, Illumina) and subjected to 100SE sequencing on a Hiseq 2000 sequencer (Illumina, Parque Científico de Madrid, PCM), resulting in 20 million reads per sample.

### Sequencing data processing and alignment for small RNAs.

Strand-specific, single-end reads between 18 nt and 42 nt, provided by BGI sequencing services, were quality-checked with FASTQC (www.bioinformatics.babraham.ac.uk/projects/fastqc/). No additional filters were necessary. Short reads were aligned against mouse genome sequence (GRCm38) with BWA (86) allowing up to 1 mismatch and no gaps (bwa aln -n 1 -k 1 -o 0). Only unique hits were considered for posterior steps. SAM alignment files generated by BWA were compressed, sorted, and indexed using the “view,” “sort,” and “index” functions of the Samtools package (87).

### Definition of candidate small RNA loci data set (genomic features).

A unified list of candidate regions (small RNA loci) was created by collapsing coverage data of all 16 samples on a single combined coverage track where, for each nucleotide, the maximum individual coverage was recorded. In this track, continuous genomic regions with a combined coverage of 2 or more nucleotides and with a summit of at least 10 nt were considered. In this way, a set of 5,208 genomic features was defined, with an average size of 36 nt. Features overlapping with known mouse mmu­miRNAs from miRBase v.20 (36) were annotated. In this way, 593 out of 2,035 (29%) of all miRBase v.20 mmu­miRNAs were detected.

### Differential expression determination of small RNAs.

Illumina short reads were assigned to the genomic features defined above with the “featureCounts” program of the SubRead package (88) using default parameters for single­end, strand­specific reads. To evaluate differential expression of features between samples from SARS-CoV-ΔE and WT at 2 dpi, edgeR (89) was used. Feature counts were normalized by adjusting the trimmed mean of log2 ratios (default TMM method). Three biological replicates per sample were included and FDR adjusted P values were obtained (90).

### Sequencing data processing and alignment for long RNAs.

Raw 100 nt strand-specific single-end reads, provided by Parque Científico de Madrid sequencing services, were quality checked with FASTQC (www.bioinformatics.babraham.ac.uk/projects/fastqc/). As many samples presented undetermined nucleotides (“N”) and decreased quality after the76th position, all reads from all samples were truncated to the first 75 nucleotides. Truncated (75 nt) reads were aligned against mouse genome sequence (GRCm38) with TopHat2 (91), allowing intron sizes up to 100,000 nt and using default settings for single-end alignments. An IGV genomic browser (89) was used for visualizing read alignments.

### Differential expression determination of longRNAs.

The Htseq-count function of the HTSeq package (92) was used for assigning reads to each mouse gene and lncRNA. Genomic feature coordinates were obtained from mouse annotation version GRCm38.76 (ENSEMBL). To evaluate differential expression of features between SARS-CoV-ΔE (2 days p.i.) and wild-type (2 days p.i.) samples, DESeq2 (93) was used. Default parameters were used except detection of outlier counts by Cook’s distance, which was not applied (cooksCutoff=FALSE). Three biological replicates per sample were included in the analysis, and *P* values obtained were adjusted by FDR (90). FIESTA tool (http://bioinfogp.cnb.csic.es/tools/FIESTA) was used to visualize differential expression results for genes, applying such statistical parameters as fold change and FDR (Table 2).

### Searching for targets.

The search of targets for annotated miRNAs was performed using the advanced search of miRTarBase (https://mirtarbase.cuhk.edu.cn/~miRTarBase/miRTarBase_2022/php/index.php) (39), which allows to load several miRNAs at the same time. In a first search, miRNAs upregulated in lungs infected with the attenuated ΔE versus the virulent WT virus at 2 dpi were loaded, and in a second search, the downregulated miRNAs (FDR < 0.05 and |FC| > 2) (Table 1). Those miRNA targets for up- or downregulated miRNAs validated with strong evidence using reporter assay, Western blot, or qPCR methods were chosen to study functional and gene ontology realationships using the Panther database (http://pantherdb.org). The settings for target genes classification were Mus musculus as the reference organism, panther pathways as annotation data set, and no Bonferroni correction. The statistically enriched (*P* value < 0.1) pathways for some of the target genes were drawn as a network together with miRNAs and target genes using the Cytoscape v3.2.1 tool (94).

### Cell lines.

The mouse brain tumor cells stably expressing the murine SARS-CoV receptor Angiotensin Converting Enzyme 2 (ACE2) were generated (DBTmACE2) in our laboratory (95). Infection of DBT-mACE2 cells with SARS-CoV-MA15 yielded high virus titers and induced the expression of pro-inflammatory cytokines and IFN-β, as observed in the mouse animal model and in human patients. African green monkey kidney-derived Vero E6 cells were kindly provided by E. Snijder (Medical Center Leiden University, Netherlands). Calu-3 2B4 cells are a clonal population sorted for ACE2 expression from human airway epithelial Calu-3 cells, kindly provided by K. Tseng (Medical Center Texas University, USA). DBT-mACE2 and Vero E6 cells were maintained in Dulbecco's modified Eagle's medium (DMEM, GIBCO) supplemented with 25 mM HEPES, 2 mM l-glutamine (Sigma), 1% nonessential amino acids (Sigma), and 10% FBS (Biowhittaker). Calu-3 2B4 were grown as described above and supplemented with 20% FBS.

### Virus titration and plaque assay.

For virus titration and plaque detection, supernatants of infected cells were added to confluent monolayers of Vero E6 cells and incubated for 45 min at 37°C. Media were removed and cells were overlaid with DMEM containing 0.6% of low melting agarose and 2% FBS. At 72 h p.i., cells were fixed with 10% formaldehyde and stained with crystal violet.

### RT-qPCR analysis.

The long RNA fraction extracted from mouse lungs or cell cultures was prepared as described above, and subjected to retro-transcriptase (RT) reactions using a High-Capacity cDNA transcription kit (Applied Biosystems) to generate cDNAs. qPCR analysis of mouse mRNAs was performed using TaqMan Assays (Applied Biosystems) specific for *CCL2* (Mm00441242_m1), *IL-6* (Mm00446190_m1), *CXCL10* (Mm00445235_m1), *ISG15* (Mm01705338_s1), *CXCL2* (Mm 00436450_m1), *IL-1ß* (Mm 01336189_m1), *NLRP3* (Mm00840904_m1), *CFTR* (Mm00441638_m1), *PARP-1* (Mm01321083_m1), and *MEF2C* (Mm01340842_m1). *18S* rRNA was used as an internal control for normalization (Mm03928990_g1). The small RNA fraction extracted from mouse lungs or cell cultures was subjected to RT reactions using TaqMan MicroRNA Reverse Transcription kit (Applied Biosystems) to generate cDNAs. qPCR analysis was performed using TaqMan Micro RNA Assays (Applied Biosystems) for miRNAs *223-3p* (002295) and *145a-5p* (002278). *snRNA-U6* (001973) was used as control for normalization of miRNAs from cells or lungs. Data were acquired with an ABI Prism 7500 sequence detection system (Applied Biosystems) and analyzed using ABI Prism 7500 SDS version 1.2.3 software. Gene expression relative to mock-infected samples is shown. The relative quantifications were performed using the 2^-ΔΔCt^ method (96).

### miRNA transfection in cell culture.

Mimics of cellular miRNAs miR-223-3p and miR-145a-5p (miScript miRNA Inhibitor, Qiagen) were transfected into Calu-3 2B4 cells using a reverse transfection protocol. Cells were grown to 90% confluence on 100 mm-diameter plates in the absence of antibiotics. Twenty-five nM miRNA mimics were each mixed with 1.5 μL of TransIT-X2 (Mirus Bio LLC) in 50 μL of Optimem and incubated in 24-well plates for 30 min for complex formation, according to manufacturer’s specifications. After 6 h, transfection complexes were removed and cells were infected with either SARS-CoV-MA15-ΔE or -WT at an MOI of 1. Cell supernatants were collected at 48 and 72 h p.i. for viral titration. The long and small RNA fractions were extracted from cells at the same time points using mirVana miRNA isolation kit (Ambion) and subjected to RT-qPCR analysis.

### Construction of plasmids with miRNA target sequences.

For quantitative analysis of post-transcriptional silencing by miRNAs, luciferase reporter plasmids were constructed on pMirTarget Vectors (OriGene). Target sequences for each miRNA were chemically synthesized (GeneArt, Life Technologies) containing 10 perfectly complementary repeats separated from each other by the eight nucleotides of the *Not* I recognition sequence. DNA fragments with unique restriction sites at the ends were cloned into the multicloning site of pMirTarget Vector, at the 3′ UTR of the luciferase gene.

### Luciferase reporter assay.

Calu-3 2B4 cells were reverse transfected with 500 ng of each pMirTarget plasmid including miRNA target sequences by using 1.5 μL of TransIT-X2 (Mirus Bio LLC) as described above. After 6 h, Calu-3 2B4 cells were infected with SARS-CoV-MA15-ΔE and -WT (MOI 1). After 18 h, the growth medium was removed and cells were lysed using Luciferase Assay System (Promega) according to the manufacturer’s specifications. Firefly luciferase activity and red fluorescence from Red Fluorescence Protein (RFP) were measured using Spectramax iD3, Molecular Devices. The relative luciferase activity was calculated as the ratio of Firefly luciferase activity to the red fluorescence of RFP, used to normalize the transfection efficiency.

### Data availability.

Small and long RNA sequencing raw data reported in this publication have been deposited in NCBI's Gene Expression Omnibus (97) and are accessible through GEO Series accession numbers GSE84081 and GSE180563, respectively.

10.1128/mbio.03135-21.1 FIG S1 Quantification of miR-223-3p and 145a-5p in cell cultures infected with SARS-CoV-ΔE and SARS-CoV-WT. RT-qPCR quantification of miR-223-3p and 145a-5p in DBT-mACE2 (A) and Calu-3 2B4 (B) infected cells. miRNA levels were made relative to those in mock-infected cells, using the ΔΔCt method and snRNA-U6 as the endogenous gene for normalization. Download FIG S1, TIF file, 0.4 MB. Copyright © 2022 Morales et al. 2022 Morales et al. https://creativecommons.org/licenses/by/4.0/ This content is distributed under the terms of the Creative Commons Attribution 4.0 International license.

## References

[1] Kuiken T, Fouchier RA, Schutten M, Rimmelzwaan GF, van Amerongen G, van Riel D, Laman JD, de Jong T, van Doornum G, Lim W, Ling AE, Chan PK, Tam JS, Zambon MC, Gopal R, Drosten C, van der Werf S, Escriou N, Manuguerra J-C, Stöhr K, Peiris JSM, Osterhaus AD. 2003. Newly discovered coronavirus as the primary cause of severe acute respiratory syndrome. Lancet 362:263–270. doi:10.1016/S0140-6736(03)13967-0. 12892955 PMC7112434

[2] Perlman S, Netland J. 2009. Coronaviruses post-SARS: update on replication and pathogenesis. Nat Rev Microbiol 7:439–450. doi:10.1038/nrmicro2147. 19430490 PMC2830095

[3] Graham RL, Donaldson EF, Baric RS. 2013. A decade after SARS: strategies for controlling emerging coronaviruses. Nat Rev Microbiol 11:836–848. doi:10.1038/nrmicro3143. 24217413 PMC5147543

[4] Muller MA, Paweska JT, Leman PA, Drosten C, Grywna K, Kemp A, Braack L, Sonnenberg K, Niedrig M, Swanepoel R. 2007. Coronavirus antibodies in African bat species. Emerg Infect Dis 13:1367–1370. doi:10.3201/eid1309.070342. 18252111 PMC2857293

[5] Zaki AM, van Boheemen S, Bestebroer TM, Osterhaus AD, Fouchier RA. 2012. Isolation of a novel coronavirus from a man with pneumonia in Saudi Arabia. N Engl J Med 367:1814–1820. doi:10.1056/NEJMoa1211721. 23075143

[6] Channappanavar R, Fehr AR, Vijay R, Mack M, Zhao J, Meyerholz DK, Perlman S. 2016. Dysregulated type I interferon and inflammatory monocyte-macrophage responses cause lethal pneumonia in SARS-CoV-infected mice. Cell Host Microbe 19:181–193. doi:10.1016/j.chom.2016.01.007. 26867177 PMC4752723

[7] Blanco-Melo D, Nilsson-Payant BE, Liu WC, Uhl S, Hoagland D, Moller R, Jordan TX, Oishi K, Panis M, Sachs D, Wang TT, Schwartz RE, Lim JK, Albrecht RA, tenOever BR. 2020. Imbalanced host response to SARS-CoV-2 drives development of COVID-19. Cell 181:1036–1045.e9. doi:10.1016/j.cell.2020.04.026. 32416070 PMC7227586

[8] DeDiego ML, Alvarez E, Almazan F, Rejas MT, Lamirande E, Roberts A, Shieh WJ, Zaki SR, Subbarao K, Enjuanes L. 2007. A severe acute respiratory syndrome coronavirus that lacks the E gene is attenuated in vitro and in vivo. J Virol 81:1701–1713. doi:10.1128/JVI.01467-06. 17108030 PMC1797558

[9] DeDiego ML, Pewe L, Alvarez E, Rejas MT, Perlman S, Enjuanes L. 2008. Pathogenicity of severe acute respiratory coronavirus deletion mutants in hACE-2 transgenic mice. Virology 376:379–389. doi:10.1016/j.virol.2008.03.005. 18452964 PMC2810402

[10] Lamirande EW, DeDiego ML, Roberts A, Jackson JP, Alvarez E, Sheahan T, Shieh WJ, Zaki SR, Baric R, Enjuanes L, Subbarao K. 2008. A live attenuated SARS coronavirus is immunogenic and efficacious in golden Syrian hamsters. J Virol 82:7721–7724. doi:10.1128/JVI.00304-08. 18463152 PMC2493341

[11] Netland J, DeDiego ML, Zhao J, Fett C, Alvarez E, Nieto-Torres JL, Enjuanes L, Perlman S. 2010. Immunization with an attenuated severe acute respiratory syndrome coronavirus deleted in E protein protects against lethal respiratory disease. Virology 399:120–128. doi:10.1016/j.virol.2010.01.004. 20110095 PMC2830353

[12] DeDiego ML, Nieto-Torres JL, Regla-Nava JA, Jimenez-Guardeño JM, Fernandez-Delgado R, Fett C, Castaño-Rodriguez C, Perlman S, Enjuanes L. 2014. Inhibition of NF-κB mediated inflammation in severe acute respiratory syndrome coronavirus-infected mice increases survival. J Virol 88:913–924. doi:10.1128/JVI.02576-13. 24198408 PMC3911641

[13] Jimenez-Guardeño JM, Nieto-Torres JL, DeDiego ML, Regla-Nava JA, Fernandez-Delgado R, Castaño-Rodriguez C, Enjuanes L. 2014. The PDZ-binding motif of severe acute respiratory syndrome coronavirus envelope protein is a determinant of viral pathogenesis. PLoS Pathog 10:e1004320. doi:10.1371/journal.ppat.1004320. 25122212 PMC4133396

[14] Nieto-Torres JL, Verdia-Baguena C, Jimenez-Guardeno JM, Regla-Nava JA, Castano-Rodriguez C, Fernandez-Delgado R, Torres J, Aguilella VM, Enjuanes L. 2015. Severe acute respiratory syndrome coronavirus E protein transports calcium ions and activates the NLRP3 inflammasome. Virology 485:330–339. doi:10.1016/j.virol.2015.08.010. 26331680 PMC4619128

[15] Pugin J, Ricou B, Steinberg KP, Suter PM, Martin TR. 1996. Proinflammatory activity in bronchoalveolar lavage fluids from patients with ARDS, a prominent role for interleukin-1. Am J Respir Crit Care Med 153:1850–1856. doi:10.1164/ajrccm.153.6.8665045. 8665045

[16] Yoshikawa T, Hill T, Li K, Peters CJ, Tseng CT. 2009. Severe acute respiratory syndrome (SARS) coronavirus-induced lung epithelial cytokines exacerbate SARS pathogenesis by modulating intrinsic functions of monocyte-derived macrophages and dendritic cells. J Virol 83:3039–3048. doi:10.1128/JVI.01792-08. 19004938 PMC2655569

[17] Cauchois R, Koubi M, Delarbre D, Manet C, Carvelli J, Blasco VB, Jean R, Fouche L, Bornet C, Pauly V, Mazodier K, Pestre V, Jarrot PA, Dinarello CA, Kaplanski G. 2020. Early IL-1 receptor blockade in severe inflammatory respiratory failure complicating COVID-19. Proc Natl Acad Sci USA 117:18951–18953. doi:10.1073/pnas.2009017117. 32699149 PMC7430998

[18] Bartel DP. 2018. Metazoan MicroRNAs. Cell 173:20–51. doi:10.1016/j.cell.2018.03.006. 29570994 PMC6091663

[19] Mengardi C, Limousin T, Ricci EP, Soto-Rifo R, Decimo D, Ohlmann T. 2017. microRNAs stimulate translation initiation mediated by HCV-like IRESes. Nucleic Acids Res 45:4810–4824. doi:10.1093/nar/gkw1345. 28077561 PMC5416841

[20] Iwasaki S, Kobayashi M, Yoda M, Sakaguchi Y, Katsuma S, Suzuki T, Tomari Y. 2010. Hsc70/Hsp90 chaperone machinery mediates ATP-dependent RISC loading of small RNA duplexes. Mol Cell 39:292–299. doi:10.1016/j.molcel.2010.05.015. 20605501

[21] Bushati N, Cohen SM. 2007. microRNA functions. Annu Rev Cell Dev Biol 23:175–205. doi:10.1146/annurev.cellbio.23.090506.123406. 17506695

[22] Guo H, Ingolia NT, Weissman JS, Bartel DP. 2010. Mammalian microRNAs predominantly act to decrease target mRNA levels. Nature 466:835–840. doi:10.1038/nature09267. 20703300 PMC2990499

[23] Bartel DP. 2009. MicroRNAs: target recognition and regulatory functions. Cell 136:215–233. doi:10.1016/j.cell.2009.01.002. 19167326 PMC3794896

[24] O'Connell RM, Rao DS, Baltimore D. 2012. microRNA regulation of inflammatory responses. Annu Rev Immunol 30:295–312. doi:10.1146/annurev-immunol-020711-075013. 22224773

[25] Saetrom P, Heale BS, Snove O, Jr, Aagaard L, Alluin J, Rossi JJ. 2007. Distance constraints between microRNA target sites dictate efficacy and cooperativity. Nucleic Acids Res 35:2333–2342. doi:10.1093/nar/gkm133. 17389647 PMC1874663

[26] Chi SW, Zang JB, Mele A, Darnell RB. 2009. Argonaute HITS-CLIP decodes microRNA-mRNA interaction maps. Nature 460:479–486. doi:10.1038/nature08170. 19536157 PMC2733940

[27] Peter ME. 2010. Targeting of mRNAs by multiple miRNAs: the next step. Oncogene 29:2161–2164. doi:10.1038/onc.2010.59. 20190803

[28] O'Neill LA, Sheedy FJ, McCoy CE. 2011. MicroRNAs: the fine-tuners of Toll-like receptor signalling. Nat Rev Immunol 11:163–175. doi:10.1038/nri2957. 21331081

[29] Contreras J, Rao DS. 2012. MicroRNAs in inflammation and immune responses. Leukemia 26:404–413. doi:10.1038/leu.2011.356. 22182919

[30] Aguado LC, Schmid S, Sachs D, Shim JV, Lim JK, tenOever BR. 2015. microRNA function is limited to cytokine control in the acute response to virus infection. Cell Host Microbe 18:714–722. doi:10.1016/j.chom.2015.11.003. 26651947 PMC4683400

[31] Peng X, Gralinski L, Ferris MT, Frieman MB, Thomas MJ, Proll S, Korth MJ, Tisoncik JR, Heise M, Luo S, Schroth GP, Tumpey TM, Li C, Kawaoka Y, Baric RS, Katze MG. 2011. Integrative deep sequencing of the mouse lung transcriptome reveals differential expression of diverse classes of small RNAs in response to respiratory virus infection. mBio 2:e00198-11. doi:10.1128/mBio.00198-11. 22086488 PMC3221602

[32] Garcia DM, Baek D, Shin C, Bell GW, Grimson A, Bartel DP. 2011. Weak seed-pairing stability and high target-site abundance decrease the proficiency of lsy-6 and other microRNAs. Nat Struct Mol Biol 18:1139–1146. doi:10.1038/nsmb.2115. 21909094 PMC3190056

[33] Fang X, Fukuda N, Barbry P, Sartori C, Verkman AS, Matthay MA. 2002. Novel role for CFTR in fluid absorption from the distal airspaces of the lung. J Gen Physiol 119:199–207. doi:10.1085/jgp.119.2.199. 11815669 PMC2233804

[34] Li X, Vargas Buonfiglio LG, Adam RJ, Stoltz DA, Zabner J, Comellas AP. 2017. Cystic fibrosis transmembrane conductance regulator potentiation as a therapeutic strategy for pulmonary edema: a proof-of-concept study in pigs. Crit Care Med 45:e1240–e1246. doi:10.1097/CCM.0000000000002720. 28953499 PMC5693779

[35] Weidenfeld S, Kuebler WM. 2017. Cytokine-regulation of Na(+)-K(+)-Cl(-) cotransporter 1 and cystic fibrosis transmembrane conductance regulator—potential role in pulmonary inflammation and edema formation. Front Immunol 8:393. doi:10.3389/fimmu.2017.00393. 28439270 PMC5383711

[36] Kozomara A, Griffiths-Jones S. 2014. miRBase: annotating high confidence microRNAs using deep sequencing data. Nucleic Acids Res 42:D68–D73. doi:10.1093/nar/gkt1181. 24275495 PMC3965103

[37] Neudecker V, Brodsky KS, Clambey ET, Schmidt EP, Packard TA, Davenport B, Standiford TJ, Weng T, Fletcher AA, Barthel L, Masterson JC, Furuta GT, Cai C, Blackburn MR, Ginde AA, Graner MW, Janssen WJ, Zemans RL, Evans CM, Burnham EL, Homann D, Moss M, Kreth S, Zacharowski K, Henson PM, Eltzschig HK. 2017. Neutrophil transfer of miR-223 to lung epithelial cells dampens acute lung injury in mice. Sci Transl Med 9:eaah5360. doi:10.1126/scitranslmed.aah5360. 28931657 PMC5842431

[38] Neudecker V, Haneklaus M, Jensen O, Khailova L, Masterson JC, Tye H, Biette K, Jedlicka P, Brodsky KS, Gerich ME, Mack M, Robertson AAB, Cooper MA, Furuta GT, Dinarello CA, O'Neill LA, Eltzschig HK, Masters SL, McNamee EN. 2017. Myeloid-derived miR-223 regulates intestinal inflammation via repression of the NLRP3 inflammasome. J Exp Med 214:1737–1752. doi:10.1084/jem.20160462. 28487310 PMC5460990

[39] Hsu SD, Lin FM, Wu WY, Liang C, Huang WC, Chan WL, Tsai WT, Chen GZ, Lee CJ, Chiu CM, Chien CH, Wu MC, Huang CY, Tsou AP, Huang HD. 2011. miRTarBase: a database curates experimentally validated microRNA-target interactions. Nucleic Acids Res 39:D163–D169. doi:10.1093/nar/gkq1107. 21071411 PMC3013699

[40] Mi H, Muruganujan A, Casagrande JT, Thomas PD. 2013. Large-scale gene function analysis with the PANTHER classification system. Nat Protoc 8:1551–1566. doi:10.1038/nprot.2013.092. 23868073 PMC6519453

[41] Chen J, Subbarao K. 2007. The Immunobiology of SARS*. Annu Rev Immunol 25:443–472. doi:10.1146/annurev.immunol.25.022106.141706. 17243893

[42] Smits SL, de Lang A, van den Brand JM, Leijten LM, van IWF, Eijkemans MJ, van Amerongen G, Kuiken T, Andeweg AC, Osterhaus AD, Haagmans BL. 2010. Exacerbated innate host response to SARS-CoV in aged non-human primates. PLoS Pathog 6:e1000756. doi:10.1371/journal.ppat.1000756. 20140198 PMC2816697

[43] DeDiego ML, Nieto-Torres JL, Jimenez-Guardeño JM, Regla-Nava JA, Castaño-Rodriguez C, Fernandez-Delgado R, Usera F, Enjuanes L. 2014. Coronavirus virulence genes with main focus on SARS-CoV envelope gene. Virus Res 194:124–137. doi:10.1016/j.virusres.2014.07.024. 25093995 PMC4261026

[44] DeDiego ML, Nieto-Torres JL, Jimenez-Guardeño JM, Regla-Nava JA, Alvarez E, Oliveros JC, Zhao J, Fett C, Perlman S, Enjuanes L. 2011. Severe acute respiratory syndrome coronavirus envelope protein regulates cell stress response and apoptosis. PLoS Pathog 7:e1002315. doi:10.1371/journal.ppat.1002315. 22028656 PMC3197621

[45] Boxberger N, Hecker M, Zettl UK. 2019. Dysregulation of inflammasome priming and activation by MicroRNAs in human immune-mediated diseases. J Immunol 202:2177–2187. doi:10.4049/jimmunol.1801416. 30962309

[46] Zhou W, Pal AS, Hsu AY, Gurol T, Zhu X, Wirbisky-Hershberger SE, Freeman JL, Kasinski AL, Deng Q. 2018. MicroRNA-223 suppresses the canonical NF-κB Pathway in basal keratinocytes to dampen neutrophilic inflammation. Cell Rep 22:1810–1823. doi:10.1016/j.celrep.2018.01.058. 29444433 PMC5839657

[47] O'Brien J, Hayder H, Zayed Y, Peng C. 2018. Overview of MicroRNA biogenesis, mechanisms of actions, and circulation. Front Endocrinol (Lausanne) 9:402. doi:10.3389/fendo.2018.00402. 30123182 PMC6085463

[48] Nieto-Torres JL, Dediego ML, Verdia-Baguena C, Jimenez-Guardeño JM, Regla-Nava JA, Fernandez-Delgado R, Castaño-Rodriguez C, Alcaraz A, Torres J, Aguilella VM, Enjuanes L. 2014. Severe acute respiratory syndrome coronavirus envelope protein ion channel activity promotes virus fitness and pathogenesis. PLoS Pathog 10:e1004077. doi:10.1371/journal.ppat.1004077. 24788150 PMC4006877

[49] Page C, Goicochea L, Matthews K, Zhang Y, Klover P, Holtzman MJ, Hennighausen L, Frieman M. 2012. Induction of alternatively activated macrophages enhances pathogenesis during severe acute respiratory syndrome coronavirus infection. J Virol 86:13334–13349. doi:10.1128/JVI.01689-12. 23015710 PMC3503056

[50] Channappanavar R, Fett C, Mack M, Ten Eyck PP, Meyerholz DK, Perlman S. 2017. Sex-based differences in susceptibility to severe acute respiratory syndrome coronavirus infection. J Immunol 198:4046–4053. doi:10.4049/jimmunol.1601896. 28373583 PMC5450662

[51] Dorhoi A, Iannaccone M, Farinacci M, Fae KC, Schreiber J, Moura-Alves P, Nouailles G, Mollenkopf HJ, Oberbeck-Muller D, Jorg S, Heinemann E, Hahnke K, Lowe D, Del Nonno F, Goletti D, Capparelli R, Kaufmann SH. 2013. MicroRNA-223 controls susceptibility to tuberculosis by regulating lung neutrophil recruitment. J Clin Invest 123:4836–4348. doi:10.1172/JCI67604. 24084739 PMC3809781

[52] Haneklaus M, Gerlic M, Kurowska-Stolarska M, Rainey AA, Pich D, McInnes IB, Hammerschmidt W, O'Neill LA, Masters SL. 2012. Cutting edge: miR-223 and EBV miR-BART15 regulate the NLRP3 inflammasome and IL-1β production. J Immunol 189:3795–3799. doi:10.4049/jimmunol.1200312. 22984081

[53] Oglesby IK, Chotirmall SH, McElvaney NG, Greene CM. 2013. Regulation of cystic fibrosis transmembrane conductance regulator by microRNA-145, -223, and -494 is altered in DeltaF508 cystic fibrosis airway epithelium. J Immunol 190:3354–3362. doi:10.4049/jimmunol.1202960. 23436935

[54] Fang X, Song Y, Hirsch J, Galietta LJ, Pedemonte N, Zemans RL, Dolganov G, Verkman AS, Matthay MA. 2006. Contribution of CFTR to apical-basolateral fluid transport in cultured human alveolar epithelial type II cells. Am J Physiol Lung Cell Mol Physiol 290:L242–L249. doi:10.1152/ajplung.00178.2005. 16143588

[55] Huppert LA, Matthay MA. 2017. Alveolar fluid clearance in pathologically relevant conditions: *in vitro* and *in vivo* models of acute respiratory distress syndrome. Front Immunol 8:371. doi:10.3389/fimmu.2017.00371. 28439268 PMC5383664

[56] Roffel MP, Bracke KR, Heijink IH, Maes T. 2020. miR-223: a key regulator in the innate immune response in asthma and COPD. Front Med (Lausanne) 7:196. doi:10.3389/fmed.2020.00196. 32509795 PMC7249736

[57] Bauernfeind F, Rieger A, Schildberg FA, Knolle PA, Schmid-Burgk JL, Hornung V. 2012. NLRP3 inflammasome activity is negatively controlled by miR-223. J Immunol 189:4175–4181. doi:10.4049/jimmunol.1201516. 22984082

[58] Yan Y, Lu K, Ye T, Zhang Z. 2019. MicroRNA-223 attenuates LPS-induced inflammation in an acute lung injury model via the NLRP3 inflammasome and TLR4/NF-κB signaling pathway via RHOB. Int J Mol Med 43:1467–1477. doi:10.3892/ijmm.2019.4075. 30747229 PMC6365085

[59] Baek D, Villen J, Shin C, Camargo FD, Gygi SP, Bartel DP. 2008. The impact of microRNAs on protein output. Nature 455:64–71. doi:10.1038/nature07242. 18668037 PMC2745094

[60] Chang YS, Ko BH, Ju JC, Chang HH, Huang SH, Lin CW. 2020. SARS unique domain (SUD) of severe acute respiratory syndrome coronavirus induces NLRP3 inflammasome-dependent CXCL10-mediated pulmonary inflammation. Int J Mol Sci 21:3179. doi:10.3390/ijms21093179. PMC7247444 32365944

[61] Roux J, McNicholas CM, Carles M, Goolaerts A, Houseman BT, Dickinson DA, Iles KE, Ware LB, Matthay MA, Pittet JF. 2013. IL-8 inhibits cAMP-stimulated alveolar epithelial fluid transport via a GRK2/PI3K-dependent mechanism. FASEB J 27:1095–1106. doi:10.1096/fj.12-219295. 23221335 PMC3574281

[62] Taganov KD, Boldin MP, Chang KJ, Baltimore D. 2006. NF-kappaB-dependent induction of microRNA miR-146, an inhibitor targeted to signaling proteins of innate immune responses. Proc Natl Acad Sci USA 103:12481–12486. doi:10.1073/pnas.0605298103. 16885212 PMC1567904

[63] Mann M, Mehta A, Zhao JL, Lee K, Marinov GK, Garcia-Flores Y, Lu LF, Rudensky AY, Baltimore D. 2017. An NF-κB-microRNA regulatory network tunes macrophage inflammatory responses. Nat Commun 8:851. doi:10.1038/s41467-017-00972-z. 29021573 PMC5636846

[64] Mehta A, Baltimore D. 2016. MicroRNAs as regulatory elements in immune system logic. Nat Rev Immunol 16:279–294. doi:10.1038/nri.2016.40. 27121651

[65] Seo GJ, Kincaid RP, Phanaksri T, Burke JM, Pare JM, Cox JE, Hsiang TY, Krug RM, Sullivan CS. 2013. Reciprocal inhibition between intracellular antiviral signaling and the RNAi machinery in mammalian cells. Cell Host Microbe 14:435–445. doi:10.1016/j.chom.2013.09.002. 24075860 PMC3837626

[66] Totura AL, Baric RS. 2012. SARS coronavirus pathogenesis: host innate immune responses and viral antagonism of interferon. Curr Opin Virol 2:264–275. doi:10.1016/j.coviro.2012.04.004. 22572391 PMC7102726

[67] Morales L, Oliveros JC, Fernandez-Delgado R, tenOever BR, Enjuanes L, Sola I. 2017. SARS-CoV-encoded small RNAs contribute to infection-associated lung pathology. Cell Host Microbe 21:344–355. doi:10.1016/j.chom.2017.01.015. 28216251 PMC5662013

[68] Samir M, Vaas LA, Pessler F. 2016. MicroRNAs in the host response to viral infections of veterinary importance. Front Vet Sci 3:86. doi:10.3389/fvets.2016.00086. 27800484 PMC5065965

[69] Landgraf P, Rusu M, Sheridan R, Sewer A, Iovino N, Aravin A, Pfeffer S, Rice A, Kamphorst AO, Landthaler M, Lin C, Socci ND, Hermida L, Fulci V, Chiaretti S, Foà R, Schliwka J, Fuchs U, Novosel A, Müller R-U, Schermer B, Bissels U, Inman J, Phan Q, Chien M, Weir DB, Choksi R, De Vita G, Frezzetti D, Trompeter H-I, Hornung V, Teng G, Hartmann G, Palkovits M, Di Lauro R, Wernet P, Macino G, Rogler CE, Nagle JW, Ju J, Papavasiliou FN, Benzing T, Lichter P, Tam W, Brownstein MJ, Bosio A, Borkhardt A, Russo JJ, Sander C, Zavolan M, et al. 2007. A mammalian microRNA expression atlas based on small RNA library sequencing. Cell 129:1401–1414. doi:10.1016/j.cell.2007.04.040. 17604727 PMC2681231

[70] Chen CZ, Li L, Lodish HF, Bartel DP. 2004. MicroRNAs modulate hematopoietic lineage differentiation. Science 303:83–86. doi:10.1126/science.1091903. 14657504

[71] Ismail N, Wang Y, Dakhlallah D, Moldovan L, Agarwal K, Batte K, Shah P, Wisler J, Eubank TD, Tridandapani S, Paulaitis ME, Piper MG, Marsh CB. 2013. Macrophage microvesicles induce macrophage differentiation and miR-223 transfer. Blood 121:984–995. doi:10.1182/blood-2011-08-374793. 23144169 PMC3567345

[72] Tabet F, Vickers KC, Cuesta Torres LF, Wiese CB, Shoucri BM, Lambert G, Catherinet C, Prado-Lourenco L, Levin MG, Thacker S, Sethupathy P, Barter PJ, Remaley AT, Rye KA. 2014. HDL-transferred microRNA-223 regulates ICAM-1 expression in endothelial cells. Nat Commun 5:3292. doi:10.1038/ncomms4292. 24576947 PMC4189962

[73] Haneklaus M, Gerlic M, O'Neill LA, Masters SL. 2013. miR-223: infection, inflammation and cancer. J Intern Med 274:215–226. doi:10.1111/joim.12099. 23772809 PMC7166861

[74] Johnnidis JB, Harris MH, Wheeler RT, Stehling-Sun S, Lam MH, Kirak O, Brummelkamp TR, Fleming MD, Camargo FD. 2008. Regulation of progenitor cell proliferation and granulocyte function by microRNA-223. Nature 451:1125–1129. doi:10.1038/nature06607. 18278031

[75] Li Y, Chan EY, Li J, Ni C, Peng X, Rosenzweig E, Tumpey TM, Katze MG. 2010. MicroRNA expression and virulence in pandemic influenza virus-infected mice. J Virol 84:3023–3032. doi:10.1128/JVI.02203-09. 20071585 PMC2826040

[76] Choi EJ, Kim HB, Baek YH, Kim EH, Pascua PN, Park SJ, Kwon HI, Lim GJ, Kim S, Kim YI, Choi YK. 2014. Differential microRNA expression following infection with a mouse-adapted, highly virulent avian H5N2 virus. BMC Microbiol 14:252. doi:10.1186/s12866-014-0252-0. 25266911 PMC4189662

[77] Bruscia EM, Zhang PX, Barone C, Scholte BJ, Homer R, Krause DS, Egan ME. 2016. Increased susceptibility of Cftr^-/-^ mice to LPS-induced lung remodeling. Am J Physiol Lung Cell Mol Physiol 310:L711–L719. doi:10.1152/ajplung.00284.2015. 26851259 PMC4836110

[78] Su X, Looney MR, Su HE, Lee JW, Song Y, Matthay MA. 2011. Role of CFTR expressed by neutrophils in modulating acute lung inflammation and injury in mice. Inflamm Res 60:619–632. doi:10.1007/s00011-011-0313-x. 21301926 PMC3116128

[79] De Palma FDE, Raia V, Kroemer G, Maiuri MC. 2020. The multifaceted roles of microRNAs in cystic fibrosis. Diagnostics (Basel) 10:1102. doi:10.3390/diagnostics10121102. PMC7765910 33348555

[80] Ramachandran S, Karp PH, Jiang P, Ostedgaard LS, Walz AE, Fisher JT, Keshavjee S, Lennox KA, Jacobi AM, Rose SD, Behlke MA, Welsh MJ, Xing Y, McCray PB. Jr. 2012. A microRNA network regulates expression and biosynthesis of wild-type and DeltaF508 mutant cystic fibrosis transmembrane conductance regulator. Proc Natl Acad Sci USA 109:13362–13367. doi:10.1073/pnas.1210906109. 22853952 PMC3421220

[81] Londino JD, Lazrak A, Noah JW, Aggarwal S, Bali V, Woodworth BA, Bebok Z, Matalon S. 2015. Influenza virus M2 targets cystic fibrosis transmembrane conductance regulator for lysosomal degradation during viral infection. FASEB J 29:2712–2725. doi:10.1096/fj.14-268755. 25795456 PMC4478808

[82] Chen L, Song W, Davis IC, Shrestha K, Schwiebert E, Sullender WM, Matalon S. 2009. Inhibition of Na+ transport in lung epithelial cells by respiratory syncytial virus infection. Am J Respir Cell Mol Biol 40:588–600. doi:10.1165/rcmb.2008-0034OC. 18952569 PMC2677438

[83] Lodge R, Ferreira Barbosa JA, Lombard-Vadnais F, Gilmore JC, Deshiere A, Gosselin A, Wiche Salinas TR, Bego MG, Power C, Routy JP, Ancuta P, Tremblay MJ, Cohen EA. 2017. Host microRNAs-221 and -222 inhibit HIV-1 entry in macrophages by targeting the CD4 viral receptor. Cell Rep 21:141–153. doi:10.1016/j.celrep.2017.09.030. 28978468

[84] Roberto VP, Tiago DM, Gautvik K, Cancela ML. 2015. Evidence for the conservation of miR-223 in zebrafish (*Danio rerio*): implications for function. Gene 566:54–62. doi:10.1016/j.gene.2015.04.022. 25869932

[85] Roberts A, Deming D, Paddock CD, Cheng A, Yount B, Vogel L, Herman BD, Sheahan T, Heise M, Genrich GL, Zaki SR, Baric R, Subbarao K. 2007. A mouse-adapted SARS-coronavirus causes disease and mortality in BALB/c mice. PLoS Pathog 3:e5. doi:10.1371/journal.ppat.0030005. 17222058 PMC1769406

[86] Li H, Durbin R. 2009. Fast and accurate short read alignment with Burrows-Wheeler transform. Bioinformatics 25:1754–1760. doi:10.1093/bioinformatics/btp324. 19451168 PMC2705234

[87] Li H, Handsaker B, Wysoker A, Fennell T, Ruan J, Homer N, Marth G, Abecasis G, Durbin R, Genome Project Data Processing S. 2009. The Sequence Alignment/Map format and SAMtools. Bioinformatics 25:2078–2079. doi:10.1093/bioinformatics/btp352. 19505943 PMC2723002

[88] Liao Y, Smyth GK, Shi W. 2014. featureCounts: an efficient general purpose program for assigning sequence reads to genomic features. Bioinformatics 30:923–930. doi:10.1093/bioinformatics/btt656. 24227677

[89] Robinson MD, McCarthy DJ, Smyth GK. 2010. edgeR: a Bioconductor package for differential expression analysis of digital gene expression data. Bioinformatics 26:139–140. doi:10.1093/bioinformatics/btp616. 19910308 PMC2796818

[90] Benjamini Y, Hochberg Y. 1995. Controlling the false discovery rate: a practical and powerful approach to multiple testing. J Roy Stat Soc B 57:289–300. doi:10.1111/j.2517-6161.1995.tb02031.x.

[91] Kim D, Pertea G, Trapnell C, Pimentel H, Kelley R, Salzberg SL. 2013. TopHat2: accurate alignment of transcriptomes in the presence of insertions, deletions and gene fusions. Genome Biol 14:R36. doi:10.1186/gb-2013-14-4-r36. 23618408 PMC4053844

[92] Anders S, Pyl PT, Huber W. 2015. HTSeq—a Python framework to work with high-throughput sequencing data. Bioinformatics 31:166–169. doi:10.1093/bioinformatics/btu638. 25260700 PMC4287950

[93] Love MI, Huber W, Anders S. 2014. Moderated estimation of fold change and dispersion for RNA-seq data with DESeq2. Genome Biol 15:550. doi:10.1186/s13059-014-0550-8. 25516281 PMC4302049

[94] Shannon P, Markiel A, Ozier O, Baliga NS, Wang JT, Ramage D, Amin N, Schwikowski B, Ideker T. 2003. Cytoscape: a software environment for integrated models of biomolecular interaction networks. Genome Res 13:2498–2504. doi:10.1101/gr.1239303. 14597658 PMC403769

[95] Regla-Nava JA, Jimenez-Guardeño JM, Nieto-Torres JL, Gallagher TM, Enjuanes L, DeDiego ML. 2013. The replication of a mouse adapted SARS-CoV in a mouse cell line stably expressing the murine SARS-CoV receptor mACE2 efficiently induces the expression of proinflammatory cytokines. J Virol Methods 193:639–646. doi:10.1016/j.jviromet.2013.07.039. 23911968 PMC3805046

[96] Livak KJ, Schmittgen TD. 2001. Analysis of relative gene expression data using real-time quantitative PCR and the 2(-Delta Delta C(T)) Method. Methods 25:402–408. doi:10.1006/meth.2001.1262. 11846609

[97] Edgar R, Domrachev M, Lash AE. 2002. Gene Expression Omnibus: NCBI gene expression and hybridization array data repository. Nucleic Acids Res 30:207–210. doi:10.1093/nar/30.1.207. 11752295 PMC99122

