# Supplementary figures and images for: Contribution of Host miRNA-223-3p to SARS-CoV-Induced Lung Inflammatory Pathology
Source: mBio. 2022 Mar 1;13(2):e03135-21. doi: 10.1128/mbio.03135-21 (PMC8941895; doi:10.1128/mbio.03135-21)

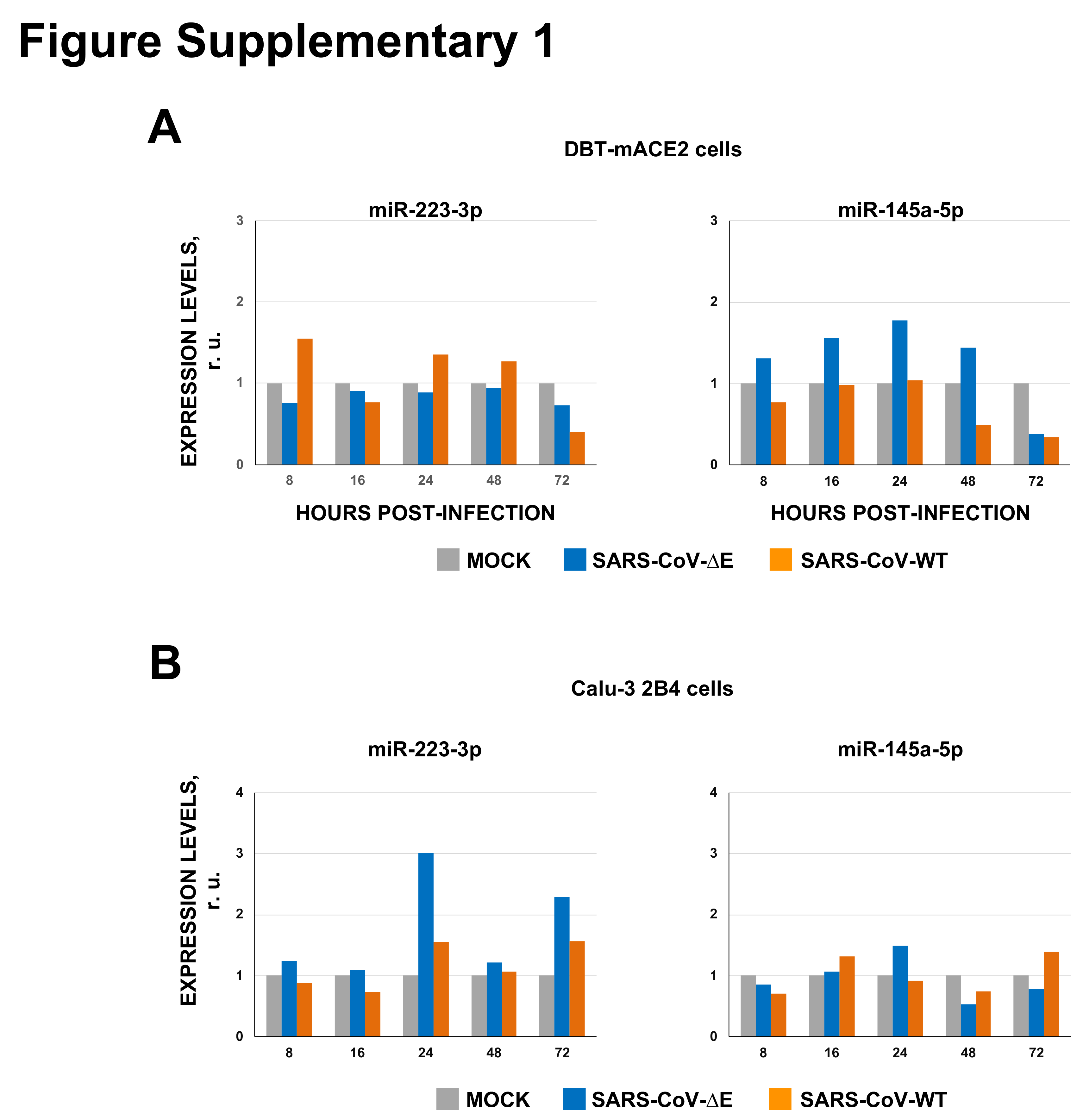

Supplement: FIG S1 [file mbio.03135-21-sf001.tif]
